# Effectiveness of insecticide thermal fogging in hyrax dens in the control of leishmaniasis vectors in rural Palestine: A prospective study

**DOI:** 10.1371/journal.pntd.0010628

**Published:** 2022-09-13

**Authors:** Samer Sawalha, Amer Al-Jawabreh, Dea Hjaija, Suheir Ereqat, Abdelmajeed Nasereddin, Hanan Al-Jawabreh, Iro Evlampidou

**Affiliations:** 1 Vector Control Unit, Environmental Health Department, Ministry of Health, Ramallah, Palestine; 2 Mediterranean and Black Sea Field Epidemiology Training Programme—MediPIET, Madrid, Spain; 3 Leishmaniases Research Unit, Jericho, Palestine; 4 Arab American University, Jenin, Palestine; 5 Preventive Medicine Department, Ministry of Health, Ramallah, Palestine; 6 Biochemistry and Molecular Biology Department, Faculty of Medicine, Al-Quds University, East Jerusalem, Palestine; Universidade Federal de Minas Gerais, BRAZIL

## Abstract

**Background:**

Zoonotic cutaneous leishmaniasis (ZCL) is endemic in Palestine and transmitted by *Phlebotomus* sand flies. They inhabit dens of hyraxes, the reservoir animal. Control measures were implemented since 1996 but cases still occur. We estimated the effect of insecticide thermal fogging inside hyrax dens on sand fly density and leishmania infection.

**Methodology/Principal findings:**

During July-September 2019, we conducted a 12-week controlled interrupted time series study in two control and one intervention sites containing three hyrax dens each. We implemented Permethrin thermal fogging in the intervention site at week 6. We measured weekly and 36hrs post-intervention sand fly abundance inside dens using CDC light traps. We performed Next-Generation Sequencing to identify sand fly *Leishmania spp*. infection. We calculated the abundance reduction (AR) using Mulla’s formula and negative binomial regression. Among 11427 collected sand flies, 7339 (64%) were females and 1786 (16%) were *Phlebotomus spp*. comprising ten species; *P*. *sergenti* was the dominant (n = 773, 43%). We report *P*. *arabicus* (n = 6) for the first time in Palestine. After fogging, *Phlebotomus spp*. AR was 93% at 36hrs, 18% and 38% at two and five weeks respectively and 41% during the complete post-intervention period. In the regression models, *Phlebotomus spp*. density in the intervention site decreased by 74% (IRR: 0.26, 95%CI: 0.11–0.57) at two weeks, 34% (IRR: 0.66, 95%CI: 0.48–0.90) at five weeks and 74% (IRR: 0.26, 95%CI: 0.12–0.59) during the complete period. The density of *Leishmania* infected sand flies decreased by 65% (IRR: 0.35, 95%CI: 0.26–0.48) at five weeks and 82% (IRR: 0.18, 95%CI: 0.07–0.42) for the complete period (zero infections until week two). *Leishmania* infection prevalence in the intervention site was 14% pre-intervention and 3.9% post-intervention.

**Conclusions/Significance:**

Fogging hyrax dens reduced sand fly abundance and leishmania infection during the 5-week post-intervention period and especially the first two weeks suggesting it could be an effective source-reduction measure for ZCL vectors. Future randomized controlled trials are needed to confirm the effectiveness of fogging hyrax dens on decreasing ZCL incidence.

## Author summary

Zoonotic cutaneous leishmaniasis (ZCL) is a neglected vector borne disease endemic in Palestine. It is transmitted by *Phlebotomus spp*. that inhabits hyrax (the main reservoir animal) dens. Despite the implementation of sanitation measures and insecticidal spraying in affected domestic areas, human cases are still reported. This suggests that complementary control measures are needed. The effectiveness of insecticide thermal fogging to control sand fly vectors inside hyrax dens has not been studied before. During 12 weeks in July-September 2019, we conducted a study in Tayasir, Tubas district (the most affected foci in northern West Bank) to estimate the effectiveness of insecticide thermal-fogging inside hyrax dens. We included one intervention and two control sites. We found a significant reduction on *Phlebotomus spp*. density and *Leishmania* infection in the intervention site at 36 hours, two and five weeks and during the whole post-intervention period. Our study findings show that insecticide thermal fogging inside hyrax is effective in reducing sand fly vectors up to five weeks post-intervention. We think that it could be an effective integrated and complementary method for source reduction of ZCL vectors especially when applied bimonthly.

## Introduction

In Palestine, three *Leishmania* species cause human disease: *Leishmania infantum*, *L*. *major* and *L*. *tropica* through a zoonotic transmission cycle involving female *Phlebotomus spp*. sand flies [1,2]. These include *P*. *sergenti*, *P*. *major* s.l., *P*. *tobbi* and *P*. *papatasi* [3,4] and all species are usually dispersed less than 500 m. *L*. *tropica* causes cutaneous leishmaniasis (CL) and is transmitted from the Rock hyrax, *Procavia capensis*, by *P*. *sergenti*. *P*. *sergenti* is more abundant inside hyrax’ habitats called dens, caves and outdoors [5–7]. Sand flies infected with *L*. *tropica* are more abundant in reservoir animals’ dens than residential and nearby areas [8]. However, in recent years, proliferation of rock-piles and other artificial shelters for hyraxes close to residential areas has resulted in their relocation here, creating new breeding sites for *P*. *sergenti* [9]. Previous studies in Palestine indicated that *L*. *tropica* infection within *P*. *sergenti* was 61% (number of tested sand flies (N) = 31) in Tubas district, 4.1% (N = 145) in Jenin District, and 1.2% (N = 162) in the Galilee region (Al-Jaleel), about 70 km north of Tubas [8,10,11].

The West Bank in Palestine is endemic to CL and visceral leishmaniasis (VL) [1,2]. In 2008–2019, the annual incidence of VL ranged between 0.01–0.2/100,000 and of CL between 7.5–13.5/100,000 with an increasing trend of zoonotic CL (ZCL) and active spread to new areas. Tubas district was among the most affected reporting 15% of all ZCL cases [12]. Most cases live on the periphery of residential areas [1].

The main ZCL control measure is to control adult sand flies through residual or space-spraying in domestic and peridomestic areas with pyrethroids [13,14]. Several field trials in residential areas in Morocco, Sudan, Libya and Greece using residual insecticides via indoor or outdoor spraying, ultra-low volume application or impregnated fine mesh, reported varying results on their effectiveness to control sand flies [15–19]. In other studies, in Panama and Iraq, thermal fogging indoors and outdoors resulted in the reduction of sand fly density but primarily in the less abundant species [20,21]. However, in desert environments, intensive use of different insecticides and application methods was not effective in controlling sand fly populations [22].

In Palestine, the *Leishmania* control programme was launched in 1996, and includes biannual indoor and outdoor residual insecticide spraying campaigns in all Leishmaniasis foci. Since 2013, spraying campaigns have increased to three per season [23]. Additional measures including small scale reservoir animal control programmes in the high risk districts of Jenin and Tubas (2012) and thermal fogging in areas of Leishmaniasis cases (since 2015) have been implemented. Despite these measures, CL cases continue to be reported which suggests the need for complementary control measures [12,24].

One of the World Health Organization (WHO) recommended methods for sand fly vector control is the fogging of hyrax habitats [25]. In this way, sand flies, including those infected with *Leishmania spp*., can be confined and eliminated inside the dens (their “source of infection” space), before emerging from the dens and spreading inhabited areas. The fogging of pre-domestic areas in CL endemic villages in Ethiopia, where vectors and reservoir hosts of CL co-exist, was suggested as an alternative to using bed nets and residual insecticide spraying for CL control [26]. However, this method has not been evaluated before.

In the last two decades, hyrax colonies in the periphery of Tayasir, Tubas district, increased markedly due to the availability of natural and artificial places that serve as their dens. Adult sand flies are present only in summer with peak activity from July to September [11,27]. Between 1990–2019, 108 CL cases (average: 6/year) with a cumulative incidence of 58/1000 (13.2% of all cases in the district) were reported in Tayasir mainly from residencies in the village periphery. During June-September 2019, we conducted a study to examine the effectiveness of thermal fogging with pyrethroids in rock hyrax dens to reducing sand fly density and *Leishmania spp*. infection in Tayasir, Tubas district, Palestine. The specific objectives of the study were to: (1) identify the sand fly species composition and estimate the sand fly density, (2) identify the *Leishmania* species and estimate the prevalence of infection in these vectors and (3) evaluate the effectiveness of thermal fogging with pyrethroids in decreasing sand fly density and *Leishmania* parasite prevalence in these vectors.

## Methods

### Study area and sand fly population

**Fig 1:**
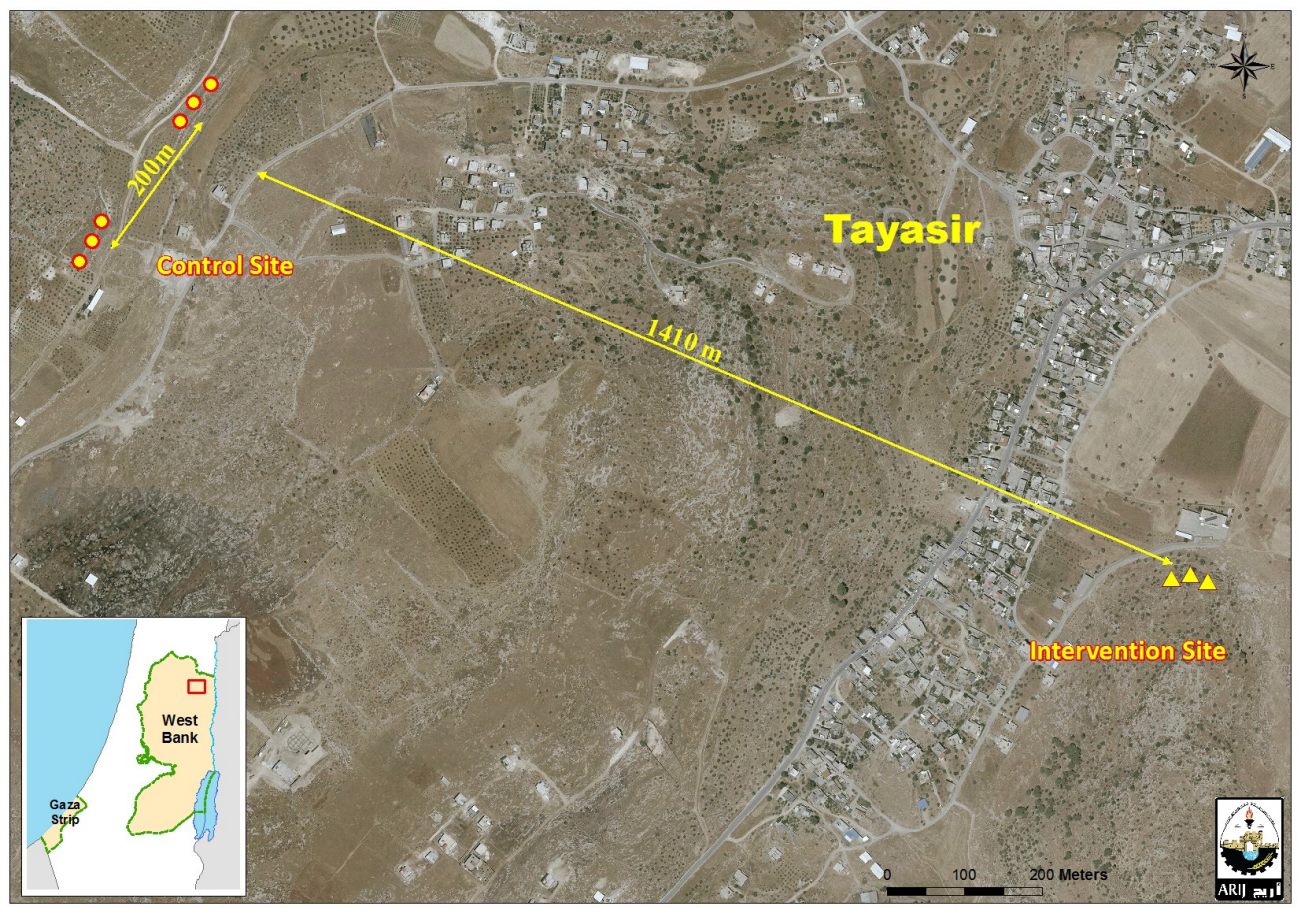
Map of Tayasir village with hyrax den locations used as control (circles) and intervention (triangles) sites. The study population consisted of the sand flies captured in the study sites during the study period. No ethical approval was obtained for this study because it did not involve any human subjects.

We conducted this study in the vicinity of Tayasir village (population: 2878) [28], Tubas district, Palestine, located 3.7 km northeast of Tubas city (Fig 1, small map bottom left). It is a rural mountainous, mainly rainfed agricultural area and a source for animal grazing with 329 mm mean annual rainfall and 21°C (range: 16.1–26.3°C) average annual temperature [29].

### Study design–Study procedure

From June to September 2019, we carried out a prospective controlled interrupted time series study [30] in Tayasir. It consisted of a baseline survey followed by a 12-week study; the later included a pre-intervention period (6 weeks), the fogging intervention and a post-intervention period (6 weeks) in one intervention and two control groups.

#### Baseline survey and allocation of control and intervention sites

**Fig 2:**
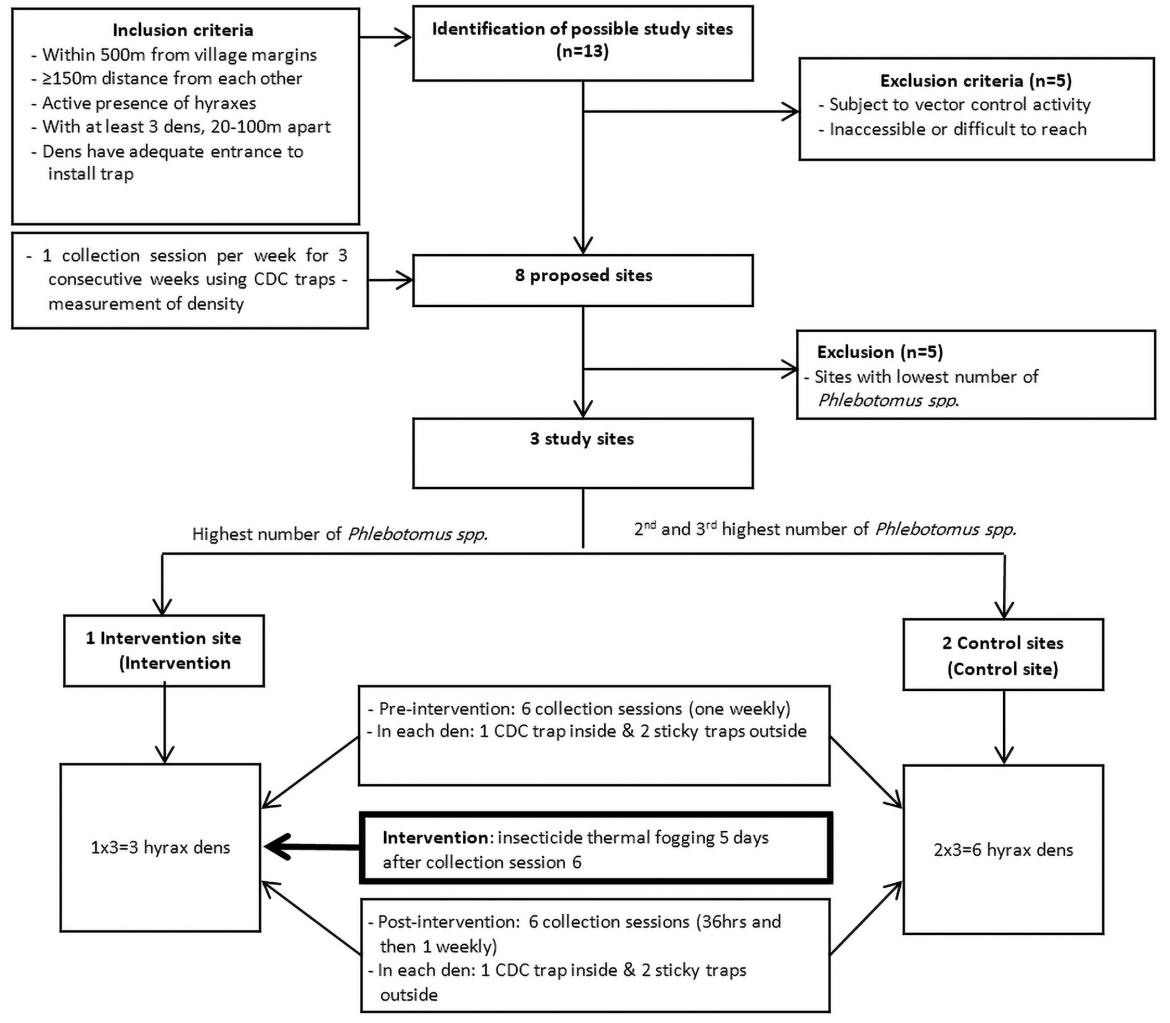
Flow diagram of study sites selection and study process, Tayasir, Palestine, 2019.

From 13–27 June 2019, we conducted a baseline survey in eight study sites (Fig 2). We defined a study site as one that was: located within 500 m from the village margins; at least 150 m apart; inhabited by rock hyraxes with a minimum of three dens 20–100 m apart; and with adequate entry openings to install the Centre for Disease Control and Prevention miniature light trap (CDC traps) (John W. Hock Co., Gainesville, FL, USA) inside the den. With the help of the local community, we initially included 13 sites fitting the inclusion criteria. We then excluded five sites as they were subject to past or planned vector control activities or because of their difficult access. Finally, eight proposed sites were included in the baseline survey.

In each proposed site, all three hyrax dens were tested, one per week, over three weeks. For the study, we selected three of eight proposed sites using the critical case sampling technique, a purposive non-probability sampling method [31]. We designated the site with the highest *Phlebotomus spp*. density as the intervention site, and the sites with the second and third highest number of *Phlebotomus spp*. density as control sites. The distance between the intervention and control sites was approximately 1400m and between the two control sites was about 200 m (Fig 1).

#### Study procedure

From 4 July to 19 September 2019, we performed 12 weekly sand fly collection sessions in all hyrax dens at the three study sites (six pre- and six post-intervention). The fogging intervention was applied in the intervention site five days after the 6^th^ collection session (pre intervention) and 36 hours before the 7^th^ (post intervention).

#### Data collection, transport and materials

For each site, we collected geographical coordinates and ecological information through direct observation using an observation sheet. We obtained meteorological data (minimum and maximum temperatures, relative humidity and wind speed) from the Palestinian Meteorological Department. We collected adult sand flies using labeled CDC and sticky paper traps as previously described [32]. During the collection sessions, the traps were always set in the same position at 17:30hrs, collected at 06:00hrs of the next day and sent to the laboratory within two hours. We installed one CDC trap as deep as possible inside every den with the trap entrance at about 50cm above the ground. During the main study period, we additionally placed two sticky traps vertically on wooden sticks at 5-20cm above the ground outside the dens’ opening at 2-5m from each side.

We performed thermal fogging according to WHO specifications using 20% Permethrin EC insecticide and fogging machine (Thermal Fogger: Shenzhen Longray Technology Co., Ltd., CN). We delivered fogging inside the hyrax dens for 1–3 minutes and outside dens at a rate of 2.5gr/1000m^3^ as described by the insecticide manufacturer and within the range of 5-10l/ha as recommended by WHO [33].

### Laboratory analysis

#### Identification of sand flies

In the laboratory, sand flies were removed from CDC collection cups after placing them at -20°C for at least 2hrs and from sticky paper traps using a fine paint brush, washed in detergent solution. They were preserved in separate labeled vials containing 70% ethanol. Each sand fly was dissected and mounted in Berlese’s medium under a binocular microscope. The head and the terminal abdominal segments were morphologically identified based on taxonomic keys [34,35]. The abdomen and thorax of gravid and engorged female *Phlebotomus spp*. were transferred to 1.5 ml Eppendorf tube containing 70% ethanol and stored at 4°C pending examination for *Leishmania spp*. infection.

#### *Leishmania* detection: Polymerase chain reaction (PCR) and next generation sequencing (NGS)

The presence and type of *Leishmania spp*. parasites in gravid and engorged female *Phlebotomus* sand flies were assessed using internal transcribed spacer 1 region (ITS1) of the ribosomal RNA gene. Briefly, the *ITS1 rRNA* gene from each specimen was amplified as previously described [6]. Primers included Illumina overhang adaptors attached to the flow cell (S2 Text). Amplification reactions were performed using X2 KAPA HiFiHotStart Ready Mix (Kappa Biosystems) with a final volume of 25μl. Negative controls containing nuclease-free water were used in each PCR run. Five μl of the amplified product were loaded on 2% agarose gel to visualize a band of ~343bp and to confirm successful amplification mainly in the positive controls (leishmanial DNA). The PCR products were purified using AMPure XP beads (X0.8) followed by a second round of amplification using the Nextera XT Index Kit (Illumina Inc, San Diego, California, USA). The prepared libraries sequenced on the Nextseq500 machine using the 150-cycle mid output kit (Illumina Inc, San Diego, California, USA).

The ITS1 rRNA gene raw sequence data were quality-filtered and analyzed using the Galaxy free online programme (https://usegalaxy.org/). Specific *Leishmania* virtual probes were used to identify each *Leishmania* species. The results were represented by the number of each species-specific reads detected. The number of reads represents the number of sequencing reactions detecting the targeted DNA. The cut-off value above which the sand fly was considered infected of harboring *Leishmania* parasite, was calculated using the ROC (Receiver operating characteristic) curve.

#### Sample size

We defined the expected prevalence of *Leishmania spp*. infection in *P*. *sergenti* at 8% (41/514) based on the results of three previous studies conducted in the broader study region [5,8,10]. Using OpenEpi version 3.01, we estimated that a sample of 83 engorged or gravid female *P*. *sergenti* (expected population size: 300) was required to detect prevalence estimates of *Leishmania* infection with 5% precision and 95% confidence level.

### Data analysis

We defined the primary outcome variable as the density of all and of *Phlebotomus spp*. sand flies. The secondary outcomes included: the density of selected *Phlebotomus* species; females; engorged or gravid; *Sergentomyia spp*.; and the total number of engorged or gravid *Phlebotomus spp*. females infected with *Leishmania spp*. in the intervention and control sites post-intervention.

To determine the similarity in the *Phlebotomus spp*. between the three sites, we calculated the Sorensen similarity coefficient that is close to 1.0 for sites with complete community overlap and close to 0.0 for completely dissimilar sites [36]. The formula is shown in S1 Text.

For each collection session, study phase and site, we calculated the: 1) relative sand fly species abundance (RA) by dividing the respective number of sand flies species by the total, 2) sand fly density by dividing the number of sand flies by the number of traps in the same site and night and 3) percentage change post-intervention. We estimated the proportion of *Leishmania spp*. infected sand flies among all tested sand flies and by species and study phase.

We performed parametric and non-parametric tests (chi2, Fisher’s exact, t-test, Wilcoxon rank-sum) to assess the statistical significance (p<0.05) when comparing study sites and the sand fly reduction post-intervention.

We estimated the percentage of sand fly abundance reduction (AR) post-intervention in the intervention and control sites for the whole post-intervention period and for each collection session separately using Mulla’s formula [37]. This formula takes into account the natural changes of sand fly density (increasing or decreasing) due to environmental factors in both intervention and control sites. The formula used is shown in S1 Text Methods.

To further evaluate the effectiveness of the fogging intervention inside the hyrax dens, for each outcome we initially explored collinearity between variables using the Pearson correlation coefficient, stationarity using the Dickey-Fuller test statistic and autocorrelations using portmanteau (Q) statistics and Bartlett’s formula for MA(q) 95% confidence bands in linear regression models.

Due to the over-dispersion and non-linearity of the data, we then performed negative binomial or Poisson regression analysis if the alpha in the negative binomial model was missing or was significantly close to 0. We used robust standard errors to account for the small number of observations and calculated incidence rate ratios (IRRs) with 95% confidence intervals (95%CI). We first did univariate analysis for each site separately and for both sites together and then built a) simple adjusted models (1a) accounting only for type of site (intervention = 1 vs. control = 0) and the fogging variable and b) fully adjusted models (1b) accounting for the fogging variable, week of collection session (trend), time after intervention (change in the trend), type of site and meteorological parameters (wind, relative humidity, minimum and maximum temperature: weekly average or at day of collection session). For a detailed description of the negative binomial and Poisson regression models see SM1. We finally reached to the most parsimonious model using backwards stepwise elimination and the Akaike’s (AIC) and Schwarz’s Bayesian (BIC) information criteria. We built secondary models (2a and 2b) in which the post-intervention phase was divided into two periods: 2 weeks and 5 weeks post-intervention (7–9 and 10–12 collection sessions, respectively). We entered data in Microsoft Excel and analyzed them in STATA 16 (StataCorp, Texas, USA).

## Results

### Study population—Sand fly species composition

During the baseline and main study (13 June-19 September 2019), we collected 13969 Phlebotomine sand flies of which 12203 (87%) from inside hyrax dens and 1766 (13%) from outside. We identified 11846 (85%) as *Sergentomyia spp*.. Fifteen percent (n = 2123) were identified as *Phlebotomus spp*. from four subgenera and ten species including *P*. (*Paraphlebotomus*) *sergenti* (972, 46%), *P*. (*Larroussius*) *major s*.*l*. (577, 27%) and *P*. (*Adlerius*) *arabicus* (7, 0.3%) (Table 1). Among all sand flies, female were 8872 (64%) and among *Phlebotomus spp*., female were 638 (30%). The male/female ratio of *Phlebotomus spp*. and *Sergentomyia spp*. was 2.3 and 0.4, respectively (p = 0.000). Among all female sand flies, 458 (5.2%) were engorged or gravid and among *Phlebotomus spp*. females, 271 (43%) were engorged or gravid.

**Table 1:** Sand fly species composition and relative abundance (RA) during the baseline survey and in the intervention (Intervention site) and control (Control site) sites during the main study period by sex, Tayasir, Palestine, June—September 2019 (N = 13969).

Genus/species	Baseline survey^a^			Intervention site ^a^			Control site^a^			Total female	%	Grand Total	RA (%)
	Female	%	Total	Female	%	Total	Female	%	Total				
***Phlebotomus spp*.**	54	26	212	509	30	1675	75	32	236	638	30	2123	15.2
*P*. *sergenti*	41	29	143	279	40	706	36	29	123	356	37	972	7.0
*P*. *major s*.*l*.	2	5	40	103	21	499	11	29	38	116	20	577	4.1
*P*. *tobbi*	8	38	21	66	18	378	12	25	48	86	19	447	3.2
*P*. *perfiliewi s*.*l*.	1	100	1	36	72	50	2	50	4	39	71	55	0.4
*P*. *papatasi*	1	100	1	3	75	4	11	58	19	15	63	24	0.2
*P*. *kazeruni*	1	100	1	12	92	13	0	0	0	13	93	14	0.1
*P*. *arabicus*	0	0	1	3	50	6	0	0	0	3	43	7	0.1
*P*. *halepensis*	0	0	1	0	0	3	0	0	0	0	0	4	0.0
*P*. *mascittii*	0	0	0	0	0	4	0	0	0	0	0	4	0.0
*P*. *alexandri*	0	0	2	0	0	0	0	0	0	0	0	2	0.0
Unidentified^b^	0	0	1	7	58	12	3	75	4	10	59	17	0.1
***Sergentomyia spp*.**	415	74	564	3251	68	4751	4568	70	6531	8234	70	11846	85
**Total**	469	74	776	3760	59	6426	4643	69	6767	8872	64	13969	100

^**a**^ Number of sites: 1) Baseline survey: 8 sites 2) Intervention: 1 site 3) Control: 2 sites

^b^ Identified only up to genus level because of specimen damage

### Baseline survey–Study site selection

During the baseline survey, among the eight candidate sites, four were natural caves and four were piles of rocks/vertical cracks in rocks (S1 Table). Among 776 collected sand flies, *Phlebotomus spp*. were 212 (27%) (Nine species, 26% females) with no significant difference in the median number between caves (median: 4, interquartile range (IQR): 3–84) and piles of rocks (median: 8, IQR: 2–17) (p = 0.661) (Table 1). The highest *Phlebotomus spp*. density was in site D (164, 78%) a natural deep cave that was designated as the intervention site. Sites A (20, 9.4%) and G (14, 6.6%) had the next highest density and they both consisted of rock piles formed by land reclamation. Sites A and G were designated as the control sites 1 and 2, respectively. The Sorensen Coefficient for control sites 1 and 2 was 0.86 with three of four same *Phlebotomus spp*. indicating very high similarity and were thus considered as one control site in the subsequent statistical analysis. The coefficient for the intervention site and control site was 0.62 indicating slight similarity with four of nine same *Phlebotomus spp*.

### Main study—Inside hyrax dens

#### Sand fly density

Among 13193 sand flies collected during the main study, 6426 (49%) were in the intervention site, of which 5715 (89%) from inside hyrax dens. Among them, 3864 (68%) were in the pre-intervention period. The mean sand fly density pre-intervention was 215 (SD: 207) and post-intervention was 103 (SD: 52) sand flies/trap/night (percent change: -52%, p = 0.749) (Table 2). Post-intervention, the percent change in the density of *Phlebotomus spp*. *was -*54% (p = 0.004), -63% (p = 0.012) for *P*. *sergenti* and -73% (p<0.001) for *P*. *major s*.*l*.

**Table 2:** Sand fly density and percent change in the intervention (Intervention site) and control (Control site) sites during the pre- and post- intervention period, Tayasir, Palestine, July-September 2019 (N = 13193).

Genus/spe*c*ies	Intervention site								Control site								*p*- value^d^
	Pre-intervention			Post-intervention			% change	*p*- value^b^	Pre-intervention			Post-intervention			% change	*p*- value^c^	
	Total	Mean	SD^a^	Total	Mean	SD^a^			Total	Mean	SD^a^	Total	Mean	SD^a^			
**Inside Hyrax dens**																	
*Phlebotomus spp*.	1088	60.4	18.0	496	27. 6	16.6	-54	0.004	114	3.2	1.1	88	2.4	0.9	-25	0.229	0.000
Females	300	16.7	3.6	199	11.1	7.3	-34	0.061	38	1.1	0.2	33	0.9	0.4	-18	0.481	0.290
Engorged or gravid	127	7.1	3.3	80	4.4	2.3	-38	0.143	16	0.4	0.4	24	0.7	0. 6	75	0.474	0.012
*Leishmania spp*. positive	17	0.9	0.4	3	0.2	0.2	-82	0.000	1	0.0	0.1	1	0.0	0.1	0.0	1.000	0.338
*P*. *sergenti*	490	27.2	13.1	180	10.0	9.0	-63	0.012	52	1.4	0.8	51	1.4	0.5	0.0	0.944	0.000
*P*. *major s*.*l*.	360	20.0	4.2	97	5.4	4.3	-73	0.000	28	0.8	0.3	4	0.1	0.1	-88	0.000	0.365
*P*. *tobbi*	188	10.4	6.0	178	9.9	5.6	-4.8	0.436	24	0.7	0.4	21	0.6	0.4	-14	0.687	0.803
*Sergentomyia spp*.	2776	154.2	191.7	1355	75.3	40.3	-51	0.748	2500	69.4	54.2	3010	83.6	29.2	21	0.262	0.000
Total sand flies	3864	214.7	206.6	1851	102.8	52.2	-52	0.749	2614	72.6	55.2	3098	86.1	29.4	19	0.262	0.000
**Outside Hyrax dens**																	
*Phlebotomus spp*.	76	4.2	2.4	15	0.8	0.6	-81	0.004	22	0.6	0.7	12	0.3	0.4	-50	0.427	0.023
Females	6	0.3	0.4	4	0.2	0.3	-33	0.300	2	0.1	0.1	2	0.1	0.1	0.0	1.000	1.000
Engorged or gravid	2	0.1	0.2	3	0.2	0.3	100	0.628	1	0.0	0.1	0	0.0	0.0	-100	0.341	1.000
*P*. *sergenti*	30	1.7	0.9	6	0.3	0.4	-82	0.005	15	0.4	0.4	5	0.1	0.3	-75	0.199	0.452
*P*. *major s*.*l*.	40	2.2	1.9	2	0.1	0.3	-96	0.003	5	0.1	0.3	1	0.0	0.1	0.0	0.461	0.336
*P*. *tobbi*	5	0.3	0.4	7	0.4	0.3	33	0.715	1	0.0	0.1	2	0.1	0.1	0.0	0.523	1.000
*Sergentomyia spp*.	340	18.9	7.6	280	15.6	4.6	-18	0.188	638	17.7	10.3	383	10.6	1.1	-40	0.124	0.002
Total sand flies	416	23.1	7.2	295	16.4	5.0	-29	0.044	660	18.3	10.2	395	11.0	0.4	-40	0.108	0.087

^**a**^ SD: Standard deviation

^b^ p-values for means in the pre- and post-intervention period in intervention site (one-tailed Student’s t-test or Wilcoxon rank-sum test)

^c^ p-values for means in the pre- and post-intervention period in control site (two-tailed Student’s t-test or Wilcoxon rank-sum test)

^d^ p-values for the totals in the pre- and post-intervention period in the Intervention site and Control site (chi-square or Fisher’s exact test)

In the control site, among 6767 collected sand flies, 5712 (84%) were from inside hyrax dens of which 2614 (46%) in the pre-intervention period. The mean sand fly density pre-intervention was 73 (SD: 55) and post-intervention was 86 (SD: 29) sand flies/trap/night (percent change: 19%, p = 0.262). Only the density of *P*. *major s*.*l*. decreased significantly post-intervention (percent change: -88%, p = 0.000).

In the intervention site, comparing to the pre-intervention period, there was a significant increase post-intervention in the proportion of *Phlebotomus spp*. females (28% vs. 40%, p = 0.000), *P*. *sergenti* (39% vs. 47%, p = 0.044), *P*. *major s*.*l*. (16% vs. 40%, p = 0.000) and *P*. *tobbi* (12% vs. 24%, p = 0.003) while in the control site, there was no significant difference (S2 Table).

#### Sand fly infection with *Leishmania spp*.

During the baseline survey and main study, among 638 *Phlebotomus spp*. females collected inside and outside dens, 271 (42%) were engorged or gravid and 249 (92%) were tested for the presence of *Leishmania* parasites. Among these, 245 (98%) were collected from inside hyrax dens of which 195 (80%) were in the intervention site during the main study (Table 3). Of 249 tested, *Leishmania* DNA was identified in 17 (6.8%) by ITS1 PCR and in 25 (10%) by NGS. The infected vector species were *P*. *sergenti* (19/135; 14%) and *P*. *major s*.*l*. (6/50; 12%). In the 25 positive specimens, two *Leishmania* species were identified by NGS: *L*. *tropica* (21, 84%) and *L*. *major* (1, 4.0%), while the rest could not be identified to species level but only as *Leishmania spp*. (3, 12%). Among 19 positive *P*. *sergenti*, 15 (79%) were infected with *L*. *tropica* (prevalence: 15/135; 11%), 3 (16%) with *Leishmania spp*. (prevalence: 3/135; 2.2%) and 1 (5.3%) with *L*. *major* (prevalence: 1/135; 0.74%). Among six positive *P*. *major s*.*l*., all (100%) were infected with *L*. *tropica* (prevalence: 6/50; 12%). DNA sequences of the *L*. *tropica* and *L*. *major* strains were deposited in the Genbank (Accession numbers: MW111284-7, MW111288-99, MW111302 and MW136330-2).

**Table 3:** Engorged or gravid female *Phlebotomus spp*. tested^a^ (N = 249) and infected (n = 25) with *Leishmania spp*. during the baseline survey and in the pre- and post-intervention period in the intervention (Intervention site) and control (Control site) sites and percent change, Tayasir, Palestine, June—September 2019.

Sandfly species	Baseline survey (8 sites)			Intervention site								Control site								p-value^d^	Total		
				Pre-intervention			Post-intervention			% change	p-value^b^	Pre-intervention			Post-intervention			% change	p-value^c^				
	N	n	%	N	n	%	N	n	%			N	n	%	N	n	%				N	n	%
***P*. *sergenti***	10	3	30.0	77^e^	14	18.2	30^f^	2	6.7	-63	0.226	9	0	0	9	0	0.0	0	1.000	0.000	135	19	14.1
***P*. *major s*.*l*.**	2	0	0.0	25^e^	3	12.0	19	1	5.3	-56	0.622	3	1	33.3	1	1	100	200	1.000	1.000	50	6	12.0
**Others** ^**g**^	2	0	0.0	19	0	0.0	29	0	0.0	0	1.000	2	0	0.0	12	0	0.0	0	1.000	1.000	64	0	0.0
**Total**	14	3	21.4	121	17	14.1	78	3	3.9	-73	0.028	14	1	7.1	22	1	4.6	-35	1.000	0.338	249	25	10.0

^a^ tested by Next-Generation Sequencing

^b^ p-values for total infected (n) in the pre- and post-intervention period in intervention site (Fisher’s exact test)

^c^ p-values for total infected (n) in the pre- and post-intervention period in control site (Fisher’s exact test)

^d^ p-values for total infected in the pre- and post-intervention period for Intervention site and Control site (Fisher’s exact test)

^e^ includes one sand fly collected outside hyrax dens

^f^ includes two sand flies collected outside hyrax dens

^g^ the category “Others” includes (in parenthesis total numbers tested): *P*. *tobbi* (35), *P*. *perfiliewi s*.*l*. (11), *P*. *kazeruni* (4), *P*. *arabicus (1)*, *P*. *papatasi (7)* and unidentified *Phlebotomus spp*. *(6)*

In the intervention site, the *Leishmania spp*. infection prevalence in *Phlebotomus spp*. was 14% (17/121) in the pre-intervention period and 3.9% (3/78) in the post-intervention period (percent change: -73%, p = 0.028). The infection prevalence in *P*. *sergenti* was 18% (14/77) pre-intervention and 6.7% (2/30) post-intervention (percent change: -63%, p = 0.226). In *P*. *major* s.l. the infection prevalence was 12% (3/25) pre-intervention and 5.3% (1/19) post-intervention (percent change: -56%, p = 0.622) (Table 3). In the control site, one infected sand fly was collected in each study period (1/14; 7.1% pre-intervention and 1/22; 4.6% post-intervention, percent change: -35%, p = 1.000).

### Effectiveness of thermal fogging intervention

#### Intervention site: Last pre-intervention vs. first post-intervention collection sessions

**Fig 3:**
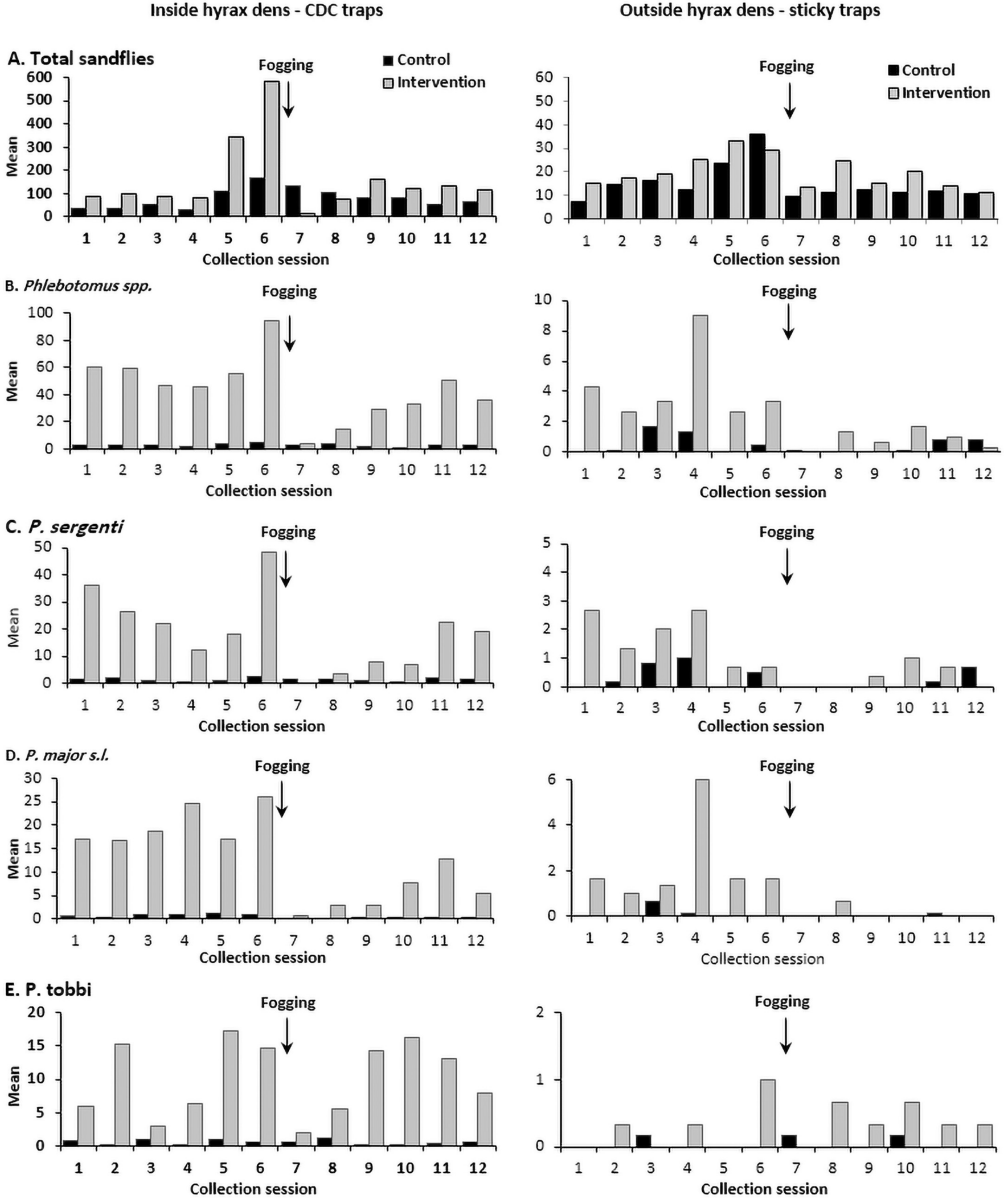
**Density of all sand flies (A), *Phlebotomus spp*. (B), *P*. *sergenti* (C), *P*. *major s*.*l*. (D) and *P*. *tobbi* (E) collected inside (right) and outside (left) hyrax dens in the intervention (Intervention site) and control (Control site) sites by collection session**^**a**^**, Tayasir, Palestine, June—September 2019 (S1 Data).** ^a^ All collection sessions occurred weekly. The 7^th^ collection session occurred 36 hours after the fogging intervention.

**Fig 4:**
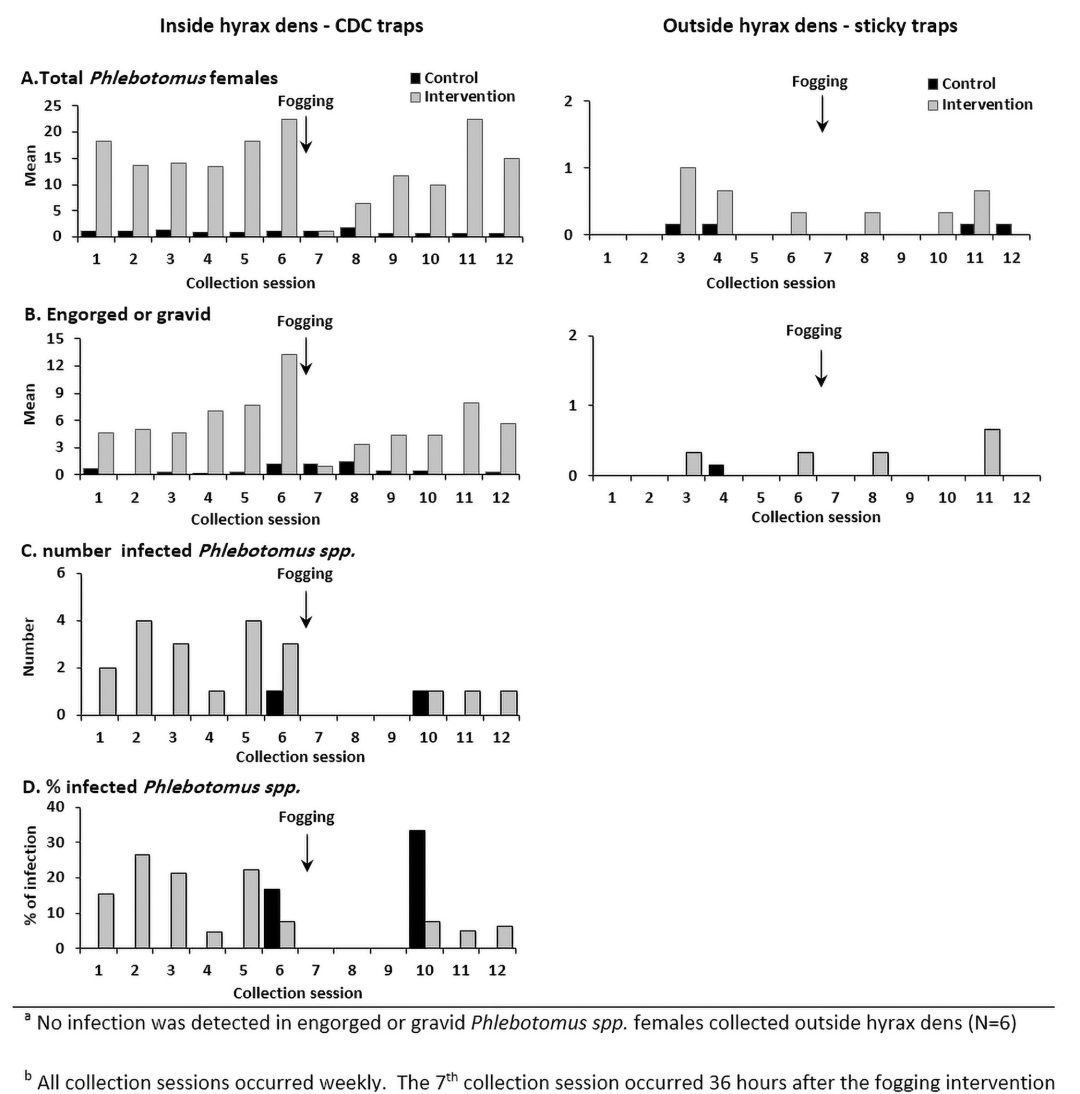
**Density of *Phlebotomus spp*. females (A), engorged or gravid females (B) and total number (C) and percentage (D) of infected females**^**a**^ **collected inside (right) and outside (left) hyrax dens in the intervention (Intervention site) and control (Control site) sites by collection session**^**b**^**, Tayasir, Palestine, June—September 2019 (S2 Data).** ^a^ No infection was detected in engorged or gravid *Phlebotomus spp*. females collected outside hyrax dens (N = 6). ^b^ All collection sessions occurred weekly. The 7^th^ collection session occurred 36 hours after the fogging intervention.

In the intervention site, the mean sand fly density at the last (6^th^) pre-intervention collection session was 581 sand flies/trap/night. This decreased by 98% (11 sand flies/trap/night) at 36 hours and by 87% (78 sand flies/trap/night) at week 1 post-intervention. The mean sand fly density of *Phlebotomus spp*. at the last (6^th^) pre-intervention session was 95 sand flies/trap/night. This decreased by 96% (3.7 sand flies/trap/night) at 36 hours and by 85% (14 sand flies/trap/night) at week 1 post-intervention (Figs 3 and 4, S3 Table, S1 and S2 Data).

In the control site, the mean sand fly density at the last (6^th^) pre-intervention was 169 sand flies/trap/night. This decreased by 22% (132 sand flies/trap/night) at 36 hours and 37% (107 sand flies/trap/night) at week 1 post-intervention. The mean sand fly density of *Phlebotomus spp*. at the last (6^th^) pre-intervention session was 4.8 sand flies/trap/night. This decreased by 42% (2.8 sand flies/trap/night) at 36 hours and 38% (3.0 sand flies/trap/night) at week 1 post-intervention.

#### Abundance reduction—Mulla’s formula

According to Mulla’s formula, in the post-intervention period in the intervention site, the abundance reduction was 60% for all sand flies, 41% for *Phlebotomus spp*. and between 24% and 63% for other outcomes (Table 4). At 36 hours and at one week post-intervention, for all outcomes the reduction was between 81% and 100% and 69% and 86%, respectively. In the following weeks, the abundance reduction was smaller, fluctuating or there was an increase as observed with *P*. *major s*.*l*., *P*. *tobbi* and *Phlebotomus spp*. females. The abundance reduction of infected *Phlebotomus spp*. sand flies in the intervention site was 94% at three weeks post-intervention, with no infection detected during the first two weeks post intervention (3 collection sessions) (Fig 4).

**Table 4:** Sand fly Abundance Reduction (%)^a^ in the intervention site (Intervention site) during the post-intervention period by collection week, Tayasir, Palestine, July-September 2019.

	Abundance Reduction (%) in Intervention site						
Characteristic	36 hours	1 Week	2 Weeks	3 Weeks	4 Weeks	5 Weeks	All period
**Inside hyrax dens**							
*Phlebotomus spp*.	93	78	18	-73	0.5	38	41
Females	95	76	-11	5.0	-112	-43	24
Engorged or gravid	95	86	45	45	- ^b^	-7.1	58
*Leishmania spp*. positive	- ^b^	- ^b^	- ^b^	94	- ^b^	- ^b^	- ^b^
*P*. *sergenti*	100	89	64	29	34	38	63
*P*. *major s*. *l*.	- ^b^	- ^b^	30	-79	-196	-24	-89
*P*. *tobbi*	81	69	-175	-526	-66	23	-8.2
*Sergentomyia spp*.	97	72	27	49	27	40	60
Total sand flies	97	75	35	49	16	38	60
**Outside hyrax dens**							
*Phlebotomus spp*.	100	- ^b^	- ^b^	-45	83	94	64
Females	- ^b^	- ^b^	- ^b^	- ^b^	33	100	33
Engorged or gravid	- ^b^	- ^b^	- ^b^	- ^b^	- ^b^	- ^b^	- ^b^
*P*. *sergenti*	- ^b^	- ^b^	- ^b^	- ^b^	0	100	40
*P*. *major*	- ^b^	- ^b^	- ^b^	- ^b^	100	- ^b^	75
*P*. *tobbi*	100	- ^b^	- ^b^	60	- ^b^	- ^b^	30
*Sergentomyia spp*.	-37	-99	-11	-54	-14	-5.3	-37
Total sand flies	-14	-78	2.2	-40	3.4	16	-19

^a^ Using Mulla’s formula: 100-((C1*T2)/C2*T1))*100 where C1 = pre-intervention mean number of sand flies in control site (C), T2 = post-intervention mean number of sand flies in intervention site, C2 = post-intervention mean number of sand flies in control site, T1 = pre-intervention mean number of sand flies in intervention site. A negative number signifies abundance increase

^b^ the indicator could not be estimated because the denominator = 0, mainly due to C2 being 0

#### Regression analysis

**Fig 5:**
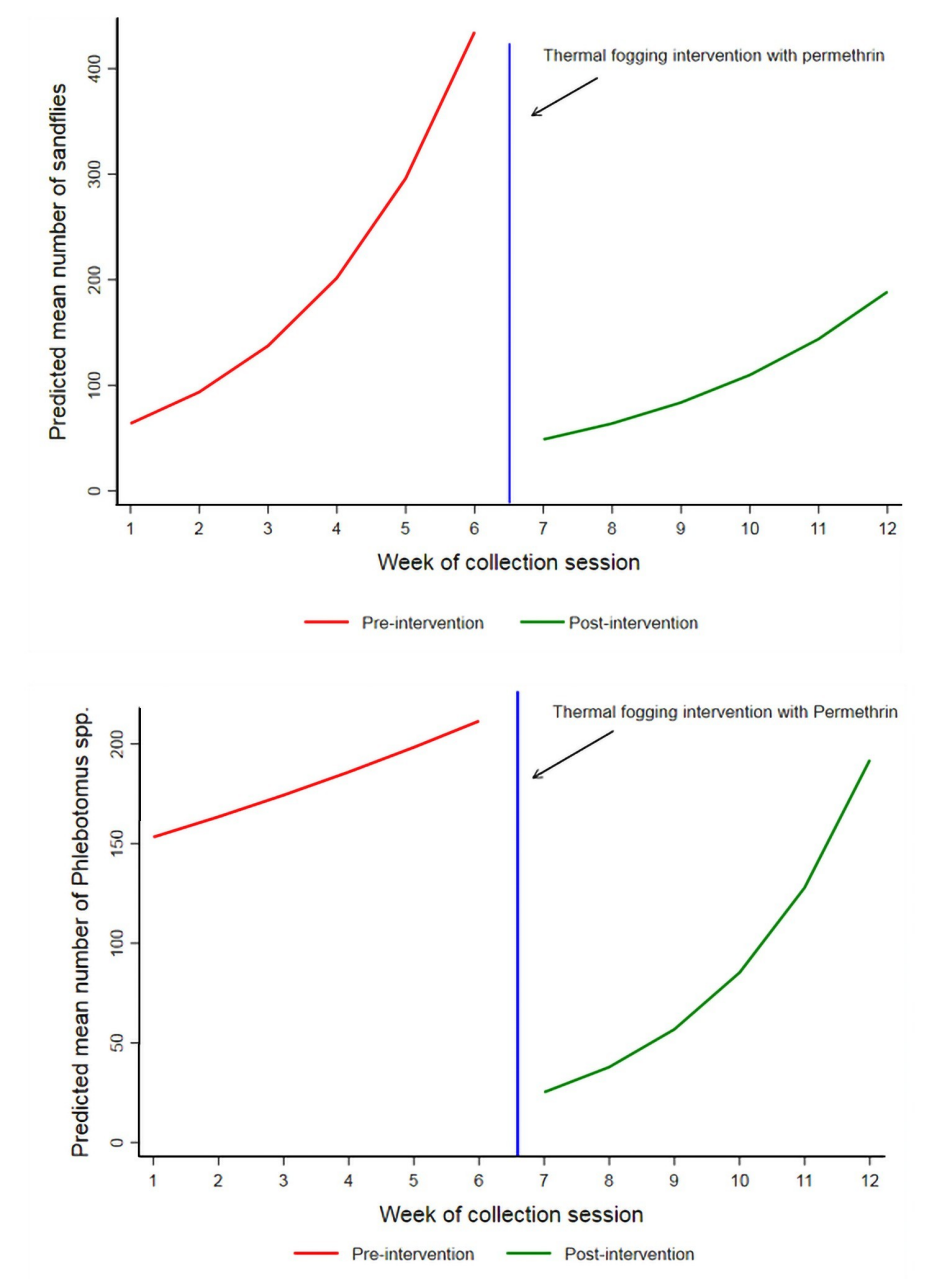
**Predicted mean number (trend) of a) all sand flies and b) *Phlebotomus spp*. sand flies in the intervention site pre- and post- intervention using negative binomial regression**^**a**^**, Tayasir, Palestine, 2019 (S3 Data).** ^a^ with robust standard errors and adjusted for the week of collection session only.

Meteorological conditions were highly correlated with each other and with the week of the collection session and time after intervention. For this reason they were excluded from the initial fully adjusted models when the latter two were significant. In the intervention site only, for total sand fly density, there was no trend for the whole study period, however there was a significant positive trend pre-intervention (IRR: 1.47, 95%CI: 1.29–1.68) and no significant trend post-intervention (IRR: 1.31, 95%CI: 0.85–2.02) (Fig 5). For *Phlebotomus spp*. there was a significant negative trend for the whole study period however there was no trend pre-intervention (IRR: 1.07, 95%CI: 0.96–1.19) and a significant positive trend post-intervention (IRR: 1.5, 95%CI: 1.10–2.04) (Fig 5).

When examining the post-intervention period as a whole, thermal fogging in the intervention site significantly reduced in both the simple (1a) and fully (1b) adjusted models the mean density of all *Phlebotomus spp*. (model 1b: 74%, IRR: 0.26, 95%CI: 0.12–0.59) and of *P*. *sergenti* (model 1b: 86%, IRR: 0.14. 95%CI: 0.05–0.38), *P*. *major s*.*l*. (1b: 71%, IRR: 0.29, 95%CI: 0.20–0.42) and the counts of *Leishmania* infected sand flies (model 1a: 82%, IRR: 0.18, 95%CI: 0.07–0.42) (Table 5 and S4 Table). The reduction was significant only in the fully adjusted model 1b for all sand flies (63%), gravid/engorged *Phlebotomus spp*. (78%), and female *Phlebotomus spp*. (73%) while *P*. *tobbi* and *Sergentomyia spp*. were unaffected.

**Table 5:** Unadjusted (uIRR) and adjusted (aIRR) Incidence Rate Ratio and 95% Confidence Intervals (95%CI) in the intervention (intervention site) and control sites (control site) for factors associated with the main and secondary outcomes in negative binomial regression^a^, Tayasir, Palestine, June—September 2019.

	Univariable						Multivariable							
	Both sites		Intervention site		Control site		Simple Adjusted model 1a^b^		Full Adjusted model 1b ^c^		Simple Adjusted model 2a^d^		Full Adjusted model 2b^e^	
Outcome/explanatory variables	uIRR	95%CI	uIRR	95%CI	uIRR	95%CI	aIRR	95%CI	aIRR	95%CI	aIRR	95%CI	aIRR	95%CI
**Total sand flies (mean)**														
**Week of collection (1–12)**	1.01	(0.94–1.10)	0.99	(0.90–1.09)	1.06	(0.96–1.16)			**1.37**	**(1.25–1.50)**			**1.39**	**(1.28–1.51)**
**Fogging (yes vs. no)**	0.83	(0.44–1.54)	0.48	(0.21–1.10)			0.48	(0.21–1.08)	**0.37**	**(0.16–0.89)**				
**Fogging period (vs. pre-intervention)**														
1–2 weeks post-intervention	0.67	(0.25–1.79)	0.39	(0.13–1.21)							0.39	(0.13–1.18)	**0.29**	**(0.11–0.82)**
3–5 weeks post-intervention	0.98	(0.60–1.62)	0.57	(0.27–1.19)							0.57	(0.28–1.17)	**0.57**	**(0.34–0.96)**
**Time after intervention (0, 1–6)** ^**f**^	0.92	(0.77–1.11)	0.92	(0.85–1.00)	0.94	(0.85–1.04)			**0.68**	**(0.55–0.85)**			**0.64**	**(0.55–0.75)**
**Intervention site (vs. control site)**	**2.00**	**(1.08–3.71)**					**2.71**	**(1.24–5.89)**	**3.06**	**(2.03–4.59)**	**2.71**	**(1.24–5.89)**	**2.89**	**(1.98–4.22)**
**R. Humidity (collection day, %)** ^**g**^	**1.05**	**(1.01–1.09)**	**1.05**	**(1.01–1.10)**	1.03	(0.99–1.08)								
**Min temperature (weekly mean,** ^**o**^**C)**^**h**^	**1.39**	**(1.00–1.93)**	1.35	(0.92–1.98)	1.47	(1.21–1.78)								
***Phlebotomus spp*.(mean)**														
**Week of collection (1–12)**	0.95	(0.85–1.06)	**0.95**	**(0.91–0.98)**	0.98	(0.94–1.02)								
**Fogging (yes vs. no)**	1.25	(0.58–2.69)	**0.46**	**(0.27–0.76)**			**0.46**	**(0.28–0.75)**	**0.26**	**(0.12–0.59)**				
**Fogging period (vs. pre-intervention)**														
1–2 weeks post-intervention	0.70	(0.26–1.88)	**0.26**	**(0.11–0.58)**							**0.26**	**(0.11–0.57)**		
3–5 weeks post-intervention	1.80	(0.93–3.48)	**0.66**	**(0.47–0.91)**							**0.66**	**(0.48–0.90)**		
**Time after intervention (0, 1–6)** ^**f**^	0.92	(0.77–1.11)	0.92	(0.85–1.00)	0.94	(0.85–1.04)			**1.14**	**(0.99–1.33)**				
**Intervention site (vs. control site)**	**15.7**	**(11.0–22.5)**					**21.5**	**(16.0–29.0)**	**27.9**	**(17.7–43.8)**	**21.5**	**(16.0–29.0)**		
**Wind speed (weekly mean, Km/h)** ^**i**^	1.79	(0.66–4.83)	1.84	(0.98–3.46)	1.27	(0.85–1.91)								
***P*. *sergenti spp*.(mean)** ^**j**^														
**Week of collection (1–12)**	0.94	(0.83–1.06)	**0.93**	**(0.87–1.00)**	1.00	(0.95–1.06)								
**Fogging (yes vs. no)**	1.00	(0.39–2.56)	**0.37**	**(0.17–0.80)**			**0.37**	**(0.17–0.79)**	**0.14**	**(0.05–0.38)**				
**Fogging period (vs. pre-intervention)**														
1–2 weeks post-intervention	0.38	(0.11–1.25)	**0.14**	**(0.05–0.41)**							**0.14**	**(0.05–0.40)**		
3–5 weeks post-intervention	1.62	(0.71–3.68)	0.60	(0.32–1.11)							0.60	(0.32–1.09)		
**Time after intervention (0, 1–6)** ^**f**^	0.92	(0.75–1.13)	0.92	(0.80–1.04)	0.99	(0.89–1.11)			**1.27**	**(1.05–1.54)**				
**Intervention site (vs. control site)**	**13.0**	**(8.03–21.1)**					**19.0**	**(12.3–29.4)**	**31.3**	**(15.1–65.3)**	**19.0**	**(12.3–29.4)**		
**Total infected females (count)** ^**j**^														
**Week of collection (1–12)**	**0.87**	**(0.77–1.00)**	**0.85**	**(0.77–0.94)**	1.14	(0.85–1.51)								
**Fogging (yes vs. no)**	0.47	(0.17–1.34)	**0.18**	**(0.07–0.43)**			**0.18**	**(0.07–0.42)**						
**Fogging period (vs. pre-intervention)**														
1–2 weeks post-intervention	0.00	(0.00–0.00)	0.00	(0.00–0.00)							**0.00**	**(0.00–0.00)**		
3–5 weeks post-intervention	0.95	(0.50–1.78)	**0.35**	**(0.26–0.48)**							**0.35**	**(0.26–0.48)**		
**Time after intervention (0, 1–6)** ^**f**^	0.80	(0.63–1.01)	**0.74**	**(0.57–0.95)**	1.05	(0.60–1.84)								
**Intervention site (vs. control site)**	**10.0**	**(2.50–39.9)**					**17.0**	**(4.50–64.2)**			**17.0**	**(4.50–64.2)**		
**Wind speed (weekly mean, Km/h)** ^**i**^	**3.26**	**(1.03–10.3)**	**3.49**	**(1.70–7.19)**	2.19	(0.15–32.8)								

^a^ With robust standard errors. When alpha too small or model did not converge, Poisson regression with robust standard errors was used instead

^b^ Adjusted for site (control site = 0, intervention site = 1) and binary fogging intervention variable (0 = control site during whole study period and intervention site pre-intervention; 1 = intervention site only post-intervention)

^c^ Adjusted for binary fogging intervention variable, time variables and non-collinear environmental variables important in the univariate analysis. When data are not shown it means that the final model was reduced to simple adjusted model 1a.

^d^ Adjusted for site and ordinal fogging intervention variable (0 = control site during whole study period and intervention site during the pre-intervention period; 1 = intervention site only for weeks 1–2 (collection sessions 7–9) post-intervention; 2 = intervention site only for weeks 3–5 (collection sessions 10–12) post-intervention)

^e^ Adjusted for ordinal fogging intervention variable, time variables and non-collinear environmental variables important in the univariate analysis. When data are not shown it means that the final model was reduced to simple adjusted model 2a

^f^ Post-intervention trend, coded as 0 for pre-intervention and 1–6 for each collection session post-intervention

^g^ Relative humidity at the day of each collection session expressed in %, per unit increase

^**h**^ Weekly mean minimum temperature in °C, per unit increase

^i^ Weekly mean wind speed in Kilometers/hour, per unit increase

^j^ Poisson regression was used for *P*. *Sergenti* (univariate for control site) and for infected sand flies (all models except univariate for both sites)

When examining the two post-intervention periods separately (weeks 2 and 5, models 2), thermal fogging in the intervention site significantly reduced in both the simple (2a) and fully adjusted (2b) models and in both sub-periods the mean density of *P*. *Major* (2b: week 2: 86%, IRR: 0.14, 95%CI: 0.07–0.28; week 5: 61%, IRR: 0.39, 95%CI: 0.33–0.47). For all sand flies, the reduction was significant in the fully adjusted model 2b only (week2: 71%, IRR: 0.29, 95%CI: 0.11–0.82; week 5: 43%, IRR: 0.57, 95%CI: 0.34–0.96) (Table 5 and S4 Table). Model 2b was reduced to simple model 2a for the count of infected sand flies (week2: 100%, IRR: 0.00, 95%CI: 0.00–0.00; week 5: 65%, IRR: 0.35, 95%CI: 0.26–0.48) and for the mean density of *Phlebotomus spp*. (week 2: 74%, IRR: 0.26, 95%CI: 0.11–0.57; week 5: 34%, IRR: 0.66, 95%CI: 0.48–0.90). The decrease was significant at 2 weeks only, for *P*. *Sergenti* (2a: 86%, IRR: 0.14, 95%CI: 0.05–0.40), for the gravid/engorged *Phlebotomus spp*. females (2b: 75%, IRR: 0.25, 95%CI: 0.12–0.52) and for the *Phlebotomus spp*. females (2b: 65%, IRR: 0.35, 95%CI: 0.17–0.69).

In all models, compared with the control site, the intervention site had significantly higher sand fly density in all outcomes of interest. In the fully adjusted models, an increasing baseline trend (pre-intervention) was observed in all sand flies, Sergentomyia spp. and gravid/engorged female *Phlebotomus spp*. while post-intervention a decreasing trend was observed in all sand flies (models 1b, 2b) and *Sergentomyia spp*., and an increasing trend in female *Phlebotomus spp*. and *P*. *sergenti*. The mean density of female *Phlebotomus spp*. was positively associated with the relative humidity at collection day and of *P*. *tobbi* with the mean weekly wind speed while *P*. *major* was negatively associated with the minimum temperature at collection day.

### Main study—Outside hyrax dens

Outside hyrax dens, the average sand fly density in the intervention site was 23 (SD: 7.2) sand flies/trap/night in the pre-intervention period and 16 (SD: 5) sand flies/trap/night in the post-intervention period (percent change: -29%, p = 0.044). In the control site, it was 18 (SD: 10) pre-intervention and 11 (SD: 0.4) sand flies/trap/night post-intervention (percent change: -40%, p = 0.108)(Table 2 and Fig 3). The percent change pre- and post-intervention in the average *Phlebotomus spp*. density in the intervention site was -80% (p = 0.004) while in the control site was -46% (p = 0.427). No *Leishmania* infection was detected in the 25 gravid/engorged females collected from outside dens. Using Mulla’s formula, we observed a 19% abundance increase in the intervention site during the whole post-intervention period and especially during the first week of the fogging (increase: 78%) (Table 4).

## Discussion

We have shown that the application of insecticide thermal fogging inside hyrax dens reduced *Leishmania* vectors at the “source of infection” during the whole post-intervention period and especially in the first two weeks. We observed that *P*. *sergenti* and other *Phlebotomus spp*. vectors of *Leishmania spp*. are abundant inside hyrax dens and collected more sand flies from caves than rock-piles. We have not identified any similar studies in the published literature to compare our findings.

We identified ten *Phlebotomus* species, most of which have been previously described in neighboring areas [27,38]. We also report the identification of *P*. *arabicus*, a CL vector found also in a nearby area of Galilee (Al-Jaleel) [8], for the first time in Palestine. The most abundant species were *P*. *sergenti*, *P*. *major s*.*l*., and *P*. *tobbi*. Some studies have confirmed that *P*. *sergenti* is more abundant inside hyrax dens, caves and outdoors compared to indoors [27,39]. All collected *Phlebotomus spp*. are known as proven or suspected vectors of *Leishmania spp*. in the region [4,5,40–42]. Consequently, hyrax dens could be considered as an important source of infection for ZCL in Tayasir.

Collected sand fly vectors in the baseline and pre-and -post intervention period were infected with *L*. *tropica* and *L*. *major*. *L*. *tropica* was the most frequently carried by *P*. *sergenti*. The proportion of *P*. *sergenti* that was infected with *L*. *tropica* in the pre-intervention phase in the intervention site was higher than that reported in previous studies: 4.1% (N = 145) in residential areas and hyrax dens in West Bank [11] and 1.2% (N = 162) in the Galilee region (Al-Jaleel), about 70 km north of Tubas [8]. In Morocco, the proportion was 3.2% (n = 216) and 3.7% (n = 273) [43,44]. There is not enough information available on the infection of *P*. *major s*.*l*. by *L*. *tropica*. The presence of *Leishmania* DNA in this species does not suggest possibility of transmission in humans as *P*. *major* might have imbibed on *L*. *tropica*-infected host at the time of trapping.

In the intervention site, we observed an increasing trend during the pre-intervention phase in the density of all sand flies, mainly *Sergentomyia spp*. and gravid or engorged *Phlebotomus spp*. This finding is similar to a study that reported the highest densities of *Phlebotomus spp*. collected close to rock crevices in August and September [7]. During the post-intervention phase, there was a decreasing trend in the density of all sand flies, mainly *Sergentomyia spp*. However, there was an increasing trend in the female *Phlebotomus spp*. and *P*. *sergenti*. Even so, thermal fogging inside the hyrax dens in the intervention site significantly decreased the density of all sand flies and especially of *Leishmania* infected *P*. *sergenti* and *P*. *major*. Additionally, there was a significant decrease in the *Phlebotomus spp*. females and the engorged/gravid ones in the post intervention phase when compared to the control sites. For most outcomes, this reduction was evident during the whole post-intervention period but was more profound during the first two weeks and could be attributed to the waning residual effect of Permethrin.

Insecticide thermal fogging has a short-lived or no residual effect [33], and Permethrin insecticide has a residual effect when used for residual spraying [13]. Accordingly, it is likely that during our study the deposition of Permethrin droplets on interior surfaces and mainly on the dens’ floor may have affected the immature stages of sand flies. This is supported by our results that we collected gravid females at 36 hours post-intervention. These gravid females would have entered the dens from outside and whereas the females present inside the den would have died as a result of the fogging. This may explain the increase post-intervention in the proportion of *Phlebotomus spp*. females in the intervention site.

Thermal fogging has a diffusion property that makes it an appropriate method to target adult sand flies that breed and rest inside caves, crevices and spaces between rock piles [33]. Its targeted application at the beginning of the sand fly season followed by two to five weeks application cycles could decrease the sand fly density and the *Leishmania* infected vectors as the season progresses. This in turn can reduce the possibility of human infection. The fogging application is considered cheap and since it is limited to hyrax dens, it limits human exposure to Permethrin, while it has a moderate toxicity in other non-target organisms including hyraxes [13]. Alternative vector control methods, such as spraying with residual insecticide are more challenging as the surface and the access to hyrax dens are more challenging due to the difficulty of reaching deep and narrow places. Even so, as pyrethroids have been the primary insecticides used in the West Bank to control pests of public health importance since 2007, insecticide resistance may have developed, thus highlighting the need for further studies to evaluate insecticide resistance in sand flies [45].

Throughout the study period, we observed a higher density of all *Phlebotomus spp*. in the intervention site. We think this might be due to this site consisting of natural deep caves whereas the control site consisted of rock piles. We also identified, a higher male to female ratio pre-intervention and the presence of engorged and gravid females in the intervention site which suggests that caves may be better suited breeding and resting sites of *Phlebotomus spp*. Previous studies in hyrax habitats in the arid eastern slopes of the West Bank (ecologically similar to Tayasir), reported that both caves and artificial boulder piles were appropriate breeding and resting sites for different sand fly species, mostly *P*. *Sergenti* [9]. However, in these studies, more sand flies were collected from caves than rock-piles mainly because of the presence of more organic matter and appropriate temperature and humidity conditions [46]. In our study, there was a positive association between relative humidity on the day of collection and female *Phlebotomus spp*. density and between the average weekly wind speed and *P*. *tobbi* density. In contrast, there was a negative association between the minimum temperature at collection day and *P*. *major s*.*l*. Other studies have also attributed differences in richness and density of *Phlebotomus spp*., collected from hyrax habitats in different sites, to variations in temperature and humidity [5,47].

Our study was subject to certain limitations. The selection of study sites involved a purposive non-probability sampling. There were two main reasons for this: i) the selection of sites with the highest sand fly density to ensure maximum sand flies collection, and to increase the probability of detecting infected sand flies and ii) the time constraints and limited resources available, namely the number of CDC light traps and budget for transportation. We used only six data points before and after the fogging intervention mainly due to the short-lived effect of Permethrin and due to the limited resources available. This data series may not have been sufficient to evaluate its effect or generalize the results. Despite our use of robust confidence intervals during the regression analysis, it resulted in wide confidence intervals in some models. However, the WHO recommends that for evaluation of the efficacy of vector control interventions, at least three data points are needed before and after intervention to perform time series analysis [48]. We could not adjust for meteorological conditions in all multivariate models due to the small number of observations and consequent loss of power and the high collinearity of meteorological data with the time variables (week of collection, time after intervention). Even so, in the models that the time variable was not significant, we used alternative models including some non-collinear meteorological variables. We only were able to capture a small number of *Phlebotomus spp*. in the intervention and control sites after the intervention and test them for *Leishmania spp*. infection. This may have resulted in a lack of power to truly detect any significant changes in infection rates. The intervention and control sites were different in terms of type of habitat and partially for surrounding flora and fauna that may have resulted in the observed differences in richness and density of *Phlebotomus spp*. Although the study was implemented during the sand fly peak activity season, the small number of sand flies per collection session and inconsistent fluctuations throughout the study period made interpretations of the results difficult. *Sergentomyia spp*. were found in large numbers and included in the data analysis despite they are not important vectors of leishmaniasis. This was done in order to examine the effect of thermal fogging on all sand fly species in hyrax habitats, even though various studies on the control of sand flies excluded *Sergentomyia spp*. from data analysis [49].

The low mean density of *Phlebotomus spp*. and infected sand flies in rock piles suggests that these hyrax dens may not be the main source of ZCL infection, although the reporting of engorged, gravid and infected females indicate that more studies are needed to clarify the importance of these types of dens in ZCL infection. However, in areas of high ZCL transmission with limited resources, sand fly control inside natural caves that serve as hyrax dens can be prioritized. The presence of other 9 species of sand fly vectors and different species of *Leishmania* parasites indicate the possibility of the presence of VL that needs to be investigated further. This was the first study that evaluated the effect of thermal fogging in hyrax dens. Further studies using a randomized clinical trial design are needed to better explore the impact of fogging as a vector control tool for ZCL. In these new studies, the use of human leishmaniasis incidence as the main outcome measure would be valuable.

## Conclusion

In this part of Palestine, *P*. *sergenti* was the most abundant *Phlebotomus* species inhabiting hyrax dens and the most infected by *L*. *tropica*. This highlights the importance of hyrax dens as a source of ZCL infection. Insecticide thermal fogging inside hyrax dens effectively reduced the density of *Phlebotomus spp*. and *Leishmania* infected sand flies during a five-week period. Consequently, fogging it appears to be a promising complementary strategy in controlling ZCL foci in limited-resource settings especially when applied every two to five weeks inside hyrax dens that are otherwise difficult to control.

## Supporting information

S1 Text Data analysis and formulas. (DOCX)

S2 Text Primers included Illumina overhang adaptors (underlined) attached to the flow cell as follows: ITS1219NGSF: TCGTCGGCAGCGTCAGATGTGTATAAGAGACAGAGCTGGATCATTTTCCGATG and ITS1219NGSR: GTCTCGTGGGCTCGGAGATGTGTATAAGAGACAGATCGCGACACGTTATGTGAG. (DOCX)

S1 Table Proposed sites’ habitat description and sand fly distribution (N = 776) by sex during baseline survey, Tayasir, Palestine, June-July, 2019. (DOCX)

S2 Table Female (n) and total sand flies collected inside and outside hyrax dens in the intervention (Intervention site) and control sites (Control site) during the pre- and post- intervention period, Tayasir, Palestine, July-September 2019. (DOCX)

S3 Table Mean sand fly density in the intervention (Intervention site) and control (Control site) sites by collection session and pre- and post- intervention period, Tayasir, Palestine, July-September 2019. (DOCX)

S4 Table Unadjusted (uIRR) and adjusted (aIRR) Incidence Rate Ratio and 95% Confidence Intervals (95%CI) for factors associated with secondary study outcomes in negative binomial regression^a^, Tayasir, Palestine, June—September 2019. (DOCX)

S1 Data Density of all sand flies, *Phlebotomus spp*. *P*. *sergenti*, *P*. *major s*.*l*. and *P*. *tobbi* collected inside and outside hyrax dens in the intervention (Intervention site) and control (Control site) sites by collection session, Tayasir, Palestine, June—September 2019. (XLSX)

S2 Data Density of *Phlebotomus spp*. females, engorged or gravid females and total number and percentage of infected females collected inside and outside hyrax dens in the intervention (Intervention site) and control (Control site) sites by collection session, Tayasir, Palestine, June—September 2019. (XLSX)

S3 Data Predicted mean number (trend) of all sand flies and *Phlebotomus spp*. sand flies in the intervention site pre- and post- intervention using negative binomial regression, Tayasir, Palestine, 2019. (XLSX)
