# Supplementary material for: Effectiveness of insecticide thermal fogging in hyrax dens in the control of leishmaniasis vectors in rural Palestine: A prospective study
Source: PLoS Negl Trop Dis. 2022 Sep 13;16(9):e0010628. doi: 10.1371/journal.pntd.0010628 (PMC9469989; doi:10.1371/journal.pntd.0010628)
Supplement: S1 Text — (DOCX) [file pntd.0010628.s001.docx]

### S1. Data analysis and formulas

#### Sorensen similarity coefficient

$$Sorensen Coefficient = \frac{2c}{(a+b)}$$

Where:

a and b = number of species in site A and site B, respectively

c = number of species common to site A and site B

#### Abundance reduction

$$\text{Abundance reduction }\left( \text{AR} \right)\text{\%=100 - }\frac{\text{C1×T2}}{\text{C2×T1}}\text{ ×100}$$

Where:

C1 and C2 = mean number of sand flies pre- and post- intervention, respectively in the control group (C)

T1 and T2 = mean number of sand flies pre- and post- intervention, respectively in the intervention site (I)

#### Negative binomial and Poisson regression models

We used the following negative binomial or Poisson model for each outcome:

$${Log(Y}_{t})=\beta_{0}+\beta_{1}*{time}_{t}+\beta_{2}*{intervention}_{t}+\beta_{3}*{time after intervention}_{t}+\beta_{4}*site [+\beta_{5}*Xi]+ e_{t}$$

Where:

Y_t_ = mean number of outcome sand flies

β_0_ = Intercept, the baseline level of the mean number of sand flies in the control group

β_1_ = baseline trend & *time_t_* = time in weeks of each collection session (values: 1-12, continuous)

β_2_ = level change & *intervention_t_* = time of fogging intervention (0=before intervention, 1=after intervention in intervention site only, binary)

β_3_ = change in the trend after the fogging intervention & *time after intervention_t_* = time passed after the fogging intervention (0=pre- and 1-6=post-intervention, continuous)

β_4_ = level change & *site* = type of site (0=control, 1=intervention, binary)

β_5_= level change & *Xi*=meteorological conditions (weekly mean or daily mean at day of collection for wind speed, minimum and maximum temperature and relative humidity in %).

e_t_ = random variability not explained by the model
