# Supplementary material for: Effectiveness of insecticide thermal fogging in hyrax dens in the control of leishmaniasis vectors in rural Palestine: A prospective study
Source: PLoS Negl Trop Dis. 2022 Sep 13;16(9):e0010628. doi: 10.1371/journal.pntd.0010628 (PMC9469989; doi:10.1371/journal.pntd.0010628)
Supplement: S2 Text — (DOCX) [file pntd.0010628.s002.docx]

**S9. Primers included Illumina overhang adaptors (underlined) attached to the flow cell as follows: ITS1219NGSF: TCGTCGGCAGCGTCAGATGTGTATAAGAGACAGAGCTGGATCATTTTCCGATG and ITS1219NGSR: GTCTCGTGGGCTCGGAGATGTGTATAAGAGACAGATCGCGACACGTTATGTGAG**
