# Supplementary material for: Effectiveness of insecticide thermal fogging in hyrax dens in the control of leishmaniasis vectors in rural Palestine: A prospective study
Source: PLoS Negl Trop Dis. 2022 Sep 13;16(9):e0010628. doi: 10.1371/journal.pntd.0010628 (PMC9469989; doi:10.1371/journal.pntd.0010628)
Supplement: S1 Table — (DOCX) [file pntd.0010628.s003.docx]

## S1 Table.

| **Site** | **Coordinates** | **Alt^a^** | **Habitat type^b^** | **Topography** | **Land use** | **Vegetation** | **Animals^c^** | **Human houses at (m)** | ***Phlebotomus spp.*** | | | | **All** | |
| --- | --- | --- | --- | --- | --- | --- | --- | --- | --- | --- | --- | --- | --- | --- |
|  |  |  |  |  |  |  |  |  | **Species** | **N** | **Female** | **%** | **N** | **%** |
| **A^c^** | **32°20'30" N 35°23'06" E** | **281** | **Pile of rocks** | **Flat land** | **Orchard, grain fields** | **Pine trees, Silybum, seasonal plants** | **Rodents** | **311** | ***P.sergenti, P.major, P.tobbi, P.alexandri,*** | **20** | **10** | **50** | **161** | **21** |
| **B** | 32°20'37" N 35°23'13" E | 283 | Natural caves/narrow entrance | Sloping rocky area | Natural lands | Sarcopoterium, Silybum, seasonal plants | Dogs, cows, sheep, rodents | 187 | *P.tobbi* | 2 | 1 | 50 | 57 | 7 |
| **C** | 32°20'34" N 35°23'26" E | 270 | Vertical cracks in rocks | Rocks with crevices | Natural lands | Carob tree, Sarcopoterium, Silybum, seasonal plants | Dogs, rodents | 73 | *P.sergenti* | 1 | 0 | 0 | 20 | 3 |
| **D^c^** | **32°20'12" N 35°23'55" E** | **314** | **Natural caves** | **Down slope mountain** | **Pastures, grain fields** | **Carob, olive, fig trees, Sarcopoterium, Silybum, seasonal plants** | **Dogs, goats, sheep, rodents, wild birds** | **219** | ***P.sergenti, P.major, P.tobbi, P.alexandri, P.arabicus, P.kazeruni, P.papatasi, P.perfiliewi, P.halebiansis*** | **164** | **30** | **18** | **283** | **36** |
| **E** | 32°20'44" N 35°23'41" E | 267 | Pile of rocks | Flat area | Pastures | Olive trees, Sarcopoterium, Silybum, seasonal plants | Dogs, goats, sheep, rodents | 92 | *P.tobbi* | 2 | 1 | 50 | 31 | 4 |
| **F** | 32°20'37" N 35°23'35" E | 236 | Natural caves | Flat area | Wild lands | Olive trees, Sarcopoterium, Silybum, seasonal plants | Goats, sheep, rodents | 102 | *P. sergenti, P. major* | 4 | 2 | 50 | 125 | 16 |
| **G^c^** | **32°20'26" N 35°23'58" E** | **305** | **Pile of rocks** | **Down slope mountain** | **Wild lands** | **Olive trees, Sarcopoterium, seasonal plants** | **Sheep, rodents** | **277** | ***P.sergenti, P.major, P.tobbi*** | **14** | **9** | **64** | **68** | **9** |
| **H** | 32°19'58" N 35°23'43" E | 348 | Natural caves | Wadi | Wild lands | Pine trees, Sarcopoterium, seasonal plants | Dogs, cows, goats, sheep, rodents | 140 | *P.sergenti, P.tobbi* | 4 | 1 | 25 | 31 | 4 |
|  |  |  |  |  |  |  |  |  | **Total** | 211 | 54 | 26 | 776 | 100 |

^a^ Altitude in meters above sea level

^b^ Description of rock hyrax habitats in the site

^c^ All sites included Rock hyraxes

^d^ Sites A and G were selected as control sites and site D was selected as intervention site
