# Supplementary material for: Effectiveness of insecticide thermal fogging in hyrax dens in the control of leishmaniasis vectors in rural Palestine: A prospective study
Source: PLoS Negl Trop Dis. 2022 Sep 13;16(9):e0010628. doi: 10.1371/journal.pntd.0010628 (PMC9469989; doi:10.1371/journal.pntd.0010628)
Supplement: S2 Table — (DOCX) [file pntd.0010628.s004.docx]

## S2 Table.

| **Genus/species** | **Intervention site** | | | | | | | **Control site** | | | | | | |
| --- | --- | --- | --- | --- | --- | --- | --- | --- | --- | --- | --- | --- | --- | --- |
|  | **Pre-intervention** | | | **Post-intervention** | | | ***p*-value** | **Pre-intervention** | | | **Post-intervention** | | | ***p*-value** |
|  | **n** | **%** | **Total** | **N** | **%** | **Total** |  | **n** | **%** | **Total** | **n** | **%** | **Total** |  |
| **Inside hyrax dens** |  |  |  |  |  |  |  |  |  |  |  |  |  |  |
| ***Phlebotomus spp.*** | 300 | 27.6 | 1088 | 199 | 40.1 | 496 | 0.000 | 38 | 33.3 | 114 | 33 | 37.5 | 88 | 0.539 |
| ***P. sergenti*** | 189 | 38.6 | 490 | 85 | 47.2 | 180 | 0.044 | 18 | 34.6 | 52 | 18 | 35.3 | 51 | 0.942 |
| ***P. major s.l.*** | 59 | 16.4 | 360 | 39 | 40.2 | 97 | 0.000 | 9 | 32.1 | 28 | 1 | 25.0 | 4 | 1.000 |
| ***P. tobbi*** | 23 | 12.2 | 188 | 43 | 24.2 | 178 | 0.003 | 7 | 29.2 | 24 | 5 | 23.8 | 21 | 0.685 |
| ***Sergentomyia spp.*** | 2090 | 75.3 | 2776 | 794 | 58.6 | 1355 | 0.000 | 1740 | 69.6 | 2500 | 2145 | 71.3 | 3010 | 0.178 |
| **Total sand flies** | 2390 | 61.9 | 3864 | 993 | 53.6 | 1851 | 0.000 | 1778 | 68.0 | 2614 | 2178 | 70.3 | 3098 | 0.062 |
| **Outside hyrax dens** |  |  |  |  |  |  |  |  |  |  |  |  |  |  |
| ***Phlebotomus spp.*** | 6 | 7.9 | 76 | 4 | 26.7 | 15 | 0.056 | 2 | 9.1 | 22 | 2 | 16.7 | 12 | 0.602 |
| ***P. sergenti*** | 2 | 6.7 | 30 | 3 | 50.0 | 6 | 0.024 | 0 | 0.0 | 15 | 0 | 0.0 | 5 | - |
| ***P. major s.l.*** | 4 | 10.0 | 40 | 1 | 50.0 | 2 | 0.226 | 1 | 20.0 | 5 | 0 | 0.0 | 1 | 1.000 |
| ***P. tobbi*** | 0 | 0.0 | 5 | 0 | 0.0 | 7 | - | 0 | 0.0 | 1 | 0 | 0.0 | 2 | - |
| ***Sergentomyia spp.*** | 186 | 54.7 | 340 | 181 | 64.6 | 280 | 0.012 | 440 | 69.0 | 638 | 243 | 63.4 | 383 | 0.070 |
| **Total sand flies** | 192 | 46.2 | 416 | 185 | 62.7 | 295 | 0.000 | 442 | 67.0 | 660 | 245 | 62.0 | 395 | 0.103 |
