# Supplementary material for: Effectiveness of insecticide thermal fogging in hyrax dens in the control of leishmaniasis vectors in rural Palestine: A prospective study
Source: PLoS Negl Trop Dis. 2022 Sep 13;16(9):e0010628. doi: 10.1371/journal.pntd.0010628 (PMC9469989; doi:10.1371/journal.pntd.0010628)
Supplement: S3 Table — (DOCX) [file pntd.0010628.s005.docx]

## S3 Table.

|  | **Intervention site** | | | | | | | | | | | |
| --- | --- | --- | --- | --- | --- | --- | --- | --- | --- | --- | --- | --- |
|  | **Pre-intervention** | | | | | | **Post-intervention** | | | | | |
| **Collection session** | **1** | **2** | **3** | **4** | **5** | **6** | **7** | **8** | **9** | **10** | **11** | **12** |
| **Inside Hyrax dens** |  |  |  |  |  |  |  |  |  |  |  |  |
| ***Phlebotomus spp.*** | 61 | 60 | 46 | 46 | 56 | 95 | 3.7 | 14 | 29 | 33 | 51 | 35 |
| **Females** | 18 | 14 | 14 | 13 | 18 | 22 | 1.0 | 6.3 | 12 | 10 | 22 | 15 |
| **Engorged or gravid** | 4.7 | 5.0 | 4.7 | 7.0 | 7.7 | 13 | 1.0 | 3.3 | 4.3 | 4.3 | 8.0 | 5.7 |
| ***Leishmania* DNA** | 0.7 | 1.3 | 1.0 | 0.3 | 1.3 | 1.0 | 0.0 | 0.0 | 0.0 | 0.3 | 0.3 | 0.3 |
| ***P. sergenti*** | 36 | 26 | 22 | 12 | 18 | 48 | 0.0 | 3.3 | 8.0 | 6.7 | 23 | 19 |
| ***P. major s.l.*** | 17 | 17 | 19 | 25 | 17 | 26 | 0.7 | 3.0 | 3.0 | 7.7 | 13 | 5.3 |
| ***P. tobbi*** | 6.0 | 15 | 3.0 | 6.3 | 17 | 15 | 2.0 | 5.7 | 14 | 16 | 13 | 8.0 |
| ***Sergentomyia spp.*** | 28 | 39 | 43 | 39 | 290 | 486 | 7.7 | 64 | 132 | 88 | 80 | 79 |
| **Total sand flies** | 89 | 98 | 89 | 84 | 346 | 581 | 11 | 78 | 161 | 121 | 131 | 115 |
|  |  |  |  |  |  |  |  |  |  |  |  |  |
|  | **Control site** | | | | | | | | | | | |
|  | **Pre-intervention** | | | | | | **Post-intervention** | | | | | |
| **Collection session** | **1** | **2** | **3** | **4** | **5** | **6** | **7** | **8** | **9** | **10** | **11** | **12** |
| **Inside Hyrax dens** |  |  |  |  |  |  |  |  |  |  |  |  |
| ***Phlebotomus spp.*** | 3.0 | 2.7 | 2.7 | 1.8 | 4.0 | 4.8 | 2.8 | 3.3 | 1.8 | 1.0 | 2.7 | 3.0 |
| **Females** | 1.2 | 1.0 | 1.3 | 0.8 | 0.8 | 1.2 | 1.2 | 1.7 | 0.7 | 0.7 | 0.7 | 0.7 |
| **Engorged or gravid** | 0.7 | 0.0 | 0.3 | 0.2 | 0.3 | 1.2 | 1.2 | 1.5 | 0.5 | 0.5 | 0.0 | 0.3 |
| ***Leishmania* DNA** | 6.7 | 0.0 | 0.0 | 0.0 | 0.0 | 0.0 | 0.2 | 0.0 | 0.0 | 0.0 | 0.2 | 0.0 |
| ***P. sergenti*** | 1.5 | 2.0 | 0.8 | 0.5 | 1.2 | 2.7 | 1.7 | 1.7 | 1.2 | 0.5 | 1.8 | 1.7 |
| ***P. major s.l.*** | 0.5 | 0.3 | 0.8 | 1.0 | 1.2 | 0.8 | 0.0 | 0.0 | 0.2 | 0.2 | 0.2 | 0.2 |
| ***P. tobbi*** | 0.8 | 0.3 | 1.0 | 0.2 | 1.0 | 0.7 | 0.7 | 1.2 | 0.3 | 0.2 | 0.5 | 0.7 |
| ***Sergentomyia spp.*** | 32 | 34 | 50 | 32 | 106 | 164 | 130 | 103 | 81 | 79 | 50 | 59 |
| **Total sand flies** | 35 | 37 | 52 | 33 | 110 | 169 | 132 | 107 | 83 | 80 | 53 | 62 |
