# Supplementary material for: Effectiveness of insecticide thermal fogging in hyrax dens in the control of leishmaniasis vectors in rural Palestine: A prospective study
Source: PLoS Negl Trop Dis. 2022 Sep 13;16(9):e0010628. doi: 10.1371/journal.pntd.0010628 (PMC9469989; doi:10.1371/journal.pntd.0010628)
Supplement: S4 Table — (DOCX) [file pntd.0010628.s006.docx]

## S4 Table.

|  | **Univariable** | | | | | | **Multivariable** | | | | | | | |
| --- | --- | --- | --- | --- | --- | --- | --- | --- | --- | --- | --- | --- | --- | --- |
|  | **Both sites** | | **Intervention site** | | **Control site** | | **Simple Adjusted model 1a^b^** | | **Full Adjusted model 1b ^c^** | | **Simple Adjusted model 2a^d^** | | **Full Adjusted model 2b^e^** | |
| **Outcome/explanatory variables** | **uIRR** | **95%CI** | **uIRR** | **95%CI** | **uIRR** | **95%CI** | **RRa** | **95%CI** | **RRa** | **95%CI** | **RRa** | **95%CI** | **RRa** | **95%CI** |
| **Female *Phlebotomus spp .*(mean)** |  |  |  |  | ^f^ |  |  |  | ^f^ |  | ^f^ |  | ^f^ |  |
| **Week of collection (1-12)** | 0.99 | (0.88-1.10) | 0.99 | (0.94-1.04) | **0.96** | **(0.93-0.99)** |  |  |  |  |  |  |  |  |
| **Fogging (yes vs. no)** | 1.78 | (0.83-3.81) | 0.66 | (0.39-1.13) |  |  | 0.66 | (0.40-1.12) | **0.27** | **(0.14-0.54)** |  |  |  |  |
| **Fogging period (vs. pre-intervention)** |  |  |  |  |  |  |  |  |  |  |  |  |  |  |
| 1-2 weeks post-intervention | 1.02 | (0.38-2.73) | **0.38** | **(0.17-0.87)** |  |  |  |  |  |  | **0.38** | **(0.17-0.86)** | **0.35** | **(0.17-0.69)** |
| 3-5 weeks post-intervention | **2.54** | **(1.28-5.06)** | 0.95 | (0.63-1.43) |  |  |  |  |  |  | 0.95 | (0.63-1.42) | 0.77 | (0.52-1.14) |
| **Time after intervention (0, 1-6)^g^** | 0.99 | (0.82-1.20) | 1.00 | (0.92-1.09) | **0.92** | **(0.88-0.96)** |  |  | **1.21** | **(1.06-1.38)** |  |  |  |  |
| **Intervention site (vs. control site)** | **14.1** | **(10.3-19.1)** |  |  |  |  | **16.9** | **(13.3-21.5)** | **29.0** | **(16.0-52.9)** | **16.9** | **(13.3-21.5)** | **18.3** | **(14.1-23.7)** |
| **R. Humidity (collection day, %)^h^** | 1.01 | (0.96-1.08) | 1.02 | (0.99-1.05) | 0.99 | (0.98-1.01) |  |  | **1.03** | **(1.01-1.05)** |  |  | **1.03** | **(1.01-1.05)** |
| **Gravid or engorged female *Phlebotomus spp.* (mean)** | | |  |  | ^f^ |  | ^f^ |  | ^f^ |  | ^f^ |  | ^f^ |  |
| **Week of collection (1-12)** | 1.00 | (0.87-1.12) | 1.00 | (0.96-1.04) | 1.02 | (0.91-1.14) |  |  | **1.19** | **(1.09-1.29)** |  |  | **1.25** | **(1.14-1.36)** |
| **Fogging (yes vs. no)** | 1.63 | (0.79-3.39) | 0.63 | (0.37-1.08) |  |  | 0.63 | (0.37-1.07) | **0.22** | **(0.12-0.43)** |  |  |  |  |
| **Fogging period (vs. pre-intervention)** |  |  |  |  |  |  |  |  |  |  |  |  |  |  |
| 1-2 weeks post-intervention | 1.06 | (0.46-2.44) | **0.41** | **(0.21-0.80)** |  |  |  |  |  |  | **0.41** | **(0.21-0.79)** | **0.25** | **(0.12-0.52)** |
| 3-5 weeks post-intervention | **2.20** | **(1.12-4.36)** | 0.85 | (0.53-1.36) |  |  |  |  |  |  | 0.85 | (0.54-1.34) | 0.53 | (0.20-1.42) |
| **Time after intervention (0, 1-6)^g^** | 0.97 | (0.80-1.18) | 0.97 | (0.87-1.08) | 0.93 | (0.78-1.11) |  |  |  |  |  |  |  |  |
| **Intervention site (vs. control site)** | **10.4** | **(5.88-18.2)** |  |  |  |  | **12.7** | **(6.99-23.1)** | **24.2** | **(11.5-51.0)** | **12.7** | **(6.99-23.1)** | **16.6** | **(9.19-29.9)** |
| **R. Humidity (collection day, %)^h^** | 1.02 | (0.96-1.09) | 1.02 | (0.98-1.05) | 1.03 | (0.95-1.12) |  |  |  |  |  |  |  |  |
| ***P. major* (mean)** |  |  |  |  | ^f^ |  | ^f^ |  | ^f^ |  | ^f^ |  | ^f^ |  |
| **Week of collection (1-12)** | 0.88 | (0.77-1.01) | **0.89** | **(0.82-0.95)** | **0.86** | **(0.78-0.94)** |  |  |  |  |  |  |  |  |
| **Fogging (yes vs. no)** | 0.77 | (0.32-1.86) | **0.27** | **(0.14-0.50)** |  |  | **0.27** | **(0.15-0.50)** | **0.29** | **(0.20-0.42)** |  |  |  |  |
| **Fogging period (vs. pre-intervention)** |  |  |  |  |  |  |  |  |  |  |  |  |  |  |
| 1-2 weeks post-intervention | **0.32** | **(0.14-0.76)** | **0.11**^f^ | **(0.06-0.20)** |  |  |  |  |  |  | **0.11** | **(0.06-0.20)** | **0.14** | **(0.07-0.28)** |
| 3-5 weeks post-intervention | 1.23 | (0.57-2.64) | **0.43**^f^ | **(0.27-0.67)** |  |  |  |  |  |  | **0.43** | **(0.28-0.67)** | **0.39** | **(0.33-0.47)** |
| **Time after intervention (0, 1-6)^g^** | 0.83 | (0.67-1.03) | **0.83** | **(0.73-0.94)** | **0.67** | **(0.53-0.84)** |  |  |  |  |  |  |  |  |
| **Intervention site (vs. control site)** | **28.6** | **(15.1-53.9)** |  |  |  |  | **45.0** | **(26.4-76.8)** | **43.4** | **(26.1-72.3)** | **45.0** | **(26.4-76.8)** | **43.7** | **(26.5-72.2)** |
| **Min temperature (collection day, ^o^C)^i^** | 0.67 | (0.35-1.26) | 0.67 | (0.41-1.10) | 0.70 | (0.42-1.17) |  |  | **0.72** | **(0.59-0.87)** |  |  | **0.80** | **(0.73-0.89)** |
| ***P. tobbi* (mean)** |  |  |  |  | ^f^ |  |  |  |  |  |  |  |  |  |
| **Week of collection (1-12)** | 1.03 | (0.91-1.17) | 1.03 | (0.95-1.11) | 0.97 | (0.91-1.05) |  |  |  |  |  |  |  |  |
| **Fogging (yes vs. no)** | **2.54** | **(1.14-5.64)** | 0.95 | (0.51-1.75) |  |  | 0.95 | (0.52-1.73) | 1.48 | (0.79-2.77) |  |  |  |  |
| **Fogging period (vs. pre-intervention)** |  |  |  |  |  |  |  |  |  |  |  |  |  |  |
| 1-2 weeks post-intervention | 1.88 | (0.65-5.44) | 0.70 | (0.27-1.80) |  |  |  |  |  |  | 0.70 | (0.28-1.77) | 1.13 | (0.71-1.78) |
| 3-5 weeks post-intervention | **3.19** | **(1.51-6.76)** | 1.19 | (0.69-2.06) |  |  |  |  |  |  | 1.19 | (0.70-2.03) | **3.79** | **(2.02-7.12)** |
| **Time after intervention (0, 1-6)^g^** | 1.03 | (0.84-1.27) | 1.04 | (0.92-1.17) | 0.94 | (0.83-1.06) |  |  |  |  |  |  |  |  |
| **Intervention site (vs. control site)** | **16.3** | **(10.7-24.8)** |  |  |  |  | **16.7** | **(9.91-28.2)** | **13.5** | **(8.07-22.6)** | **16.7** | **(9.91-28.2)** | **12.3** | **(7.37-20.4)** |
| **Min temperature (weekly mean, ^o^C)^i^** | 1.07 | (0.58-1.97) | 1.06 | (0.76-1.48) | 1.30 | (0.88-1.91) |  |  |  |  |  |  |  |  |
| **Wind speed (weekly mean, Km/h)^j^** | 1.58 | (0.60-4.16) | 1.64 | (0.96-2.81) | 1.10 | (0.69-1.75) |  |  | **2.10** | **(1.25-3.53)** |  |  | **4.24** | **(2.61-6.89)** |
| **Mean *Sergentomyia* *spp*.** |  |  |  |  |  |  |  |  |  |  |  |  |  |  |
| **Week of collection (1-12)** | 1.04 | (0.94-1.16) | 1.03 | (0.86-1.24) | 1.06 | (0.96-1.17) |  |  | **1.51** | **(1.33-1.70)** |  |  | **1.53** | **(1.37-1.72)** |
| **Fogging (yes vs. no)** | 0.74 | (0.38-1.41 | 0.49 | (0.17-1.37) |  |  | 0.49 | (0.18-1.34) | 0.46 | (0.18-1.17) |  |  |  |  |
| **Fogging period (vs. pre-intervention)** |  |  |  |  |  |  |  |  |  |  |  |  |  |  |
| 1-2 weeks post-intervention | 0.66 | (0.24-1.82) | 0.44 | (0.12-1.61) |  |  |  |  |  |  | 0.44 | (0.12-1.57) | 0.38 | (0.12-1.15) |
| 3-5 weeks post-intervention | 0.81 | (0.48-1.36) | 0.54 | (0.21-1.39) |  |  |  |  |  |  | 0.54 | (0.21-1.36) | 0.68 | (0.37-1.23) |
| **Time after intervention (0, 1-6)^g^** | 0.93 | (0.80-1.07) | 0.90 | (0.74-1.10) | 0.97 | (0.86-1.10) |  |  | **0.59** | **(0.47-0.75)** |  |  |  |  |
| **Intervention site (vs. control site)** | 1.50 | (0.72-3.12) |  |  |  |  | 2.02 | (0.76-5.35) | **1.87** | **(1.11-3.15)** | 2.02 | (0.76-5.35) | **1.77** | **(1.09-2.88)** |
| **R. Humidity (collection day, %)^h^** | **1.07** | **(1.03-1.11)** | **1.09** | **(1.04-1.15)** | 1.04 | (0.99-1.08) |  |  |  |  |  |  |  |  |
| **Min temperature (weekly mean, ^o^C)^i^** | **1.55** | **(1.16-2.07)** | **1.59** | **(1.04-2.42)** | **1.49** | **(1.22-1.80)** |  |  |  |  |  |  |  |  |
| **Wind speed (weekly mean, Km/h)^j^** | **2.14** | **(1.17-3.93)** | **3.09** | **(1.32-7.26)** | 1.20 | (0.69-2.12) |  |  |  |  |  |  |  |  |

^a^ With robust standard errors.

^b^ Adjusted for site (control site=0, intervention site=1) and binary fogging intervention variable (0=control site during whole study period and intervention site pre-intervention; 1= intervention site only post-intervention)

^c^ Adjusted for binary fogging intervention variable, time variables and non-collinear environmental variables important in the univariate analysis. When data are not shown it means that the final model was reduced to simple adjusted model 1a.

^d^ Adjusted for site and ordinal fogging intervention variable (0=control site during whole study period and intervention site during the pre-intervention period; 1= intervention site only for weeks 1-2 (collection sessions 7-9) post-intervention; 2=intervention site only for weeks 3-5 (collection sessions 10-12) post-intervention)

^e^ Adjusted for ordinal fogging intervention variable, time variables and non-collinear environmental variables important in the univariate analysis. When data are not shown it means that the final model was reduced to simple adjusted model 2a

**^f^** Poisson regression with robust standard errors was used because *alpha* in negative binomial was close to 0 or the model did not converge

^g^ Post-intervention trend, coded as 0 for pre-intervention and 1-6 for each collection session post-intervention

^h^ Relative humidity the day of each collection session expressed in %, per unit increase

^i^ Minimum temperature the day of each collection session and weekly mean minimum temperature in ^o^C, per unit increase

^j^ Weekly mean wind speed in Kilometers/hour, per unit increase
